# Adherence to an anti-inflammatory diet is associated with lower Alzheimer’s disease mortality: A modifiable risk factor in a national cohort

**DOI:** 10.1016/j.tjpad.2025.100221

**Published:** 2025-06-13

**Authors:** Ching-Chi Hsu, Shiow-Ing Wang, Sebastian Yu, Eric S. Lin, James Cheng-Chung Wei

**Affiliations:** a Board of Directors, Wizcare Medical Corporation Aggregate, Taichung 404022, Taiwan; b International Intercollegiate Ph.D. Program, National Tsing Hua University, Hsinchu 300044, Taiwan; c Center for Health Data Science, Department of Medical Research, Chung Shan Medical University Hospital, Taichung 402306, Taiwan; d Department of Health Policy and Management, College of Health Care and Management, Chung Shan Medical University, Taichung 402306, Taiwan; e Department of Dermatology, College of Medicine, Kaohsiung Medical University, Kaohsiung 807378, Taiwan; f Department of Dermatology, Kaohsiung Medical University Gangshan Hospital, Kaohsiung 820111, Taiwan; g Department of Dermatology, Kaohsiung Medical University Hospital, Kaohsiung 807377, Taiwan; h Master of Public Health Program, College of Public Health, National Taiwan University, Taipei 100025, Taiwan; i Neuroscience Research Center, Kaohsiung Medical University, Kaohsiung 807378, Taiwan; j Department of Economics, National Tsing Hua University, Hsinchu 300044, Taiwan; k EMBA/MBA/MFB/MPM/HBA Programs, National Tsing Hua University, Hsinchu 300044, Taiwan; l Institute of Medicine, Chung Shan Medical University, Taichung 402306, Taiwan; m Department of Allergy, Immunology & Rheumatology, Chung Shan Medical University Hospital, Taichung 402306, Taiwan; n Graduate Institute of Integrated Medicine, China Medical University, Taichung 406040, Taiwan

**Keywords:** Brain-gut axis, Gut dysbiosis, Ketogenic diet, Neurodegeneration, Neuroinflammation

## Abstract

**Background:**

Chronic neuroinflammation contributes to Alzheimer’s disease (AD) pathogenesis, and diet is a modifiable factor influencing inflammation. The impact of an anti-inflammatory diet on AD-specific mortality remains unclear.

**Objectives:**

To examine the association between adherence to an anti-inflammatory diet (measured as the percentage of dietary energy from anti-inflammatory foods) and AD-specific mortality, as well as all-cause mortality, in a large national cohort, and to determine whether associations differ by sex or race/ethnicity.

**Methods:**

We analyzed 18,795 U.S. adults (≥18 years) from the 2007–2014 National Health and Nutrition Examination Survey. Anti-inflammatory diet adherence was defined as the percentage of total energy intake from anti-inflammatory foods, categorized as 0 %, <5 %, 5–9.99 %, or ≥10 %. Outcomes were AD-specific mortality and all-cause mortality ascertained via the National Death Index. Cox proportional hazards models were used to estimate hazard ratios (HRs) and 95 % confidence intervals (CIs) for mortality across intake categories, adjusting for demographic, lifestyle, and health factors. Analyses were stratified by sex, race/ethnicity, and age (≥45 years for AD mortality).

**Results:**

Participants with 0 % anti-inflammatory intake had a higher all-cause mortality risk (HR 3.82, 95 % CI 1.18–12.33) compared to those with ≥10 % intake. In the overall analysis, 0 % anti-inflammatory intake showed a trend of reduced AD-specific mortality although its did not reach statistical significance after full adjustment (HR 3.04, 95 % CI 0.74–12.46 vs. ≥10 % intake; p>0.05). Notably, the inverse association between anti-inflammatory diet and AD mortality emerged in subgroup analyses. Male participants and non-Hispanic White participants with 0 % intake had the highest AD mortality hazards (HR 12.83 and 3.77, respectively, vs. ≥10 % intake), indicating significant risk reductions with anti-inflammatory diet in these groups. In contrast, no significant associations were observed in female or non-White subgroups. Even a modest intake of anti-inflammatory foods (≥10 % of calories) was associated with lower AD mortality risk in the above subgroups and with lower all-cause mortality overall.

**Conclusion:**

Greater consumption of anti-inflammatory foods was associated with lower all-cause and a trend toward lower AD-specific mortality. The observed protective effects were confined to certain subpopulations (notably men and non-Hispanic Whites). Even a small portion of the diet (10 % of calories) being anti-inflammatory was linked to reduced mortality risk in these groups, suggesting that achievable dietary changes could have an impact. These findings support modifying dietary content is a practical, low-cost intervention that could mitigate neuroinflammation to reduce AD mortality risk.

## Highlights

• Low anti-inflammatory diet intake is linked to increased Alzheimer’s disease mortality.
• Dietary inflammation is identified as a modifiable risk factor for Alzheimer’s disease mortality.
• Subgroup analyses reveal sex- and race-specific heterogeneity in the association between low anti-inflammatory diet intake and Alzheimer’s disease mortality, with males and non-Hispanic Whites exhibiting greater susceptibility.
• Future research should investigate gut-brain axis mechanisms and biomarker-based dietary interventions.

## 1 Introduction

Alzheimer’s disease (AD) is a debilitating neurodegenerative disorder that burdens individuals, families, and healthcare systems worldwide [[1], [2], [3]]. As global life expectancy increases, AD prevalence is rising, underscoring the urgent need for effective prevention strategies [4,5]. Chronic neuroinflammation is now recognized as a key contributor to AD pathogenesis, promoting amyloid‐beta accumulation, tau hyperphosphorylation, and blood–brain barrier (BBB) disruption [[6], [7], [8]].

Recent studies have focused on lifestyle factors, especially diet, as modulators of AD risk [9]. Western diets rich in processed foods, refined sugars, and saturated fats exacerbate systemic inflammation and may accelerate neurodegeneration [[10], [11], [12], [13], [14]]. In contrast, anti-inflammatory diets high in fruits, vegetables, whole grains, and lean proteins may help lower inflammation and protect against AD [[15], [16], [17]].

Although evidence supports a protective role for certain dietary patterns, the underlying mechanisms remain unclear. The brain–gut axis, for instance, has emerged as a potential pathway whereby gut microbial metabolites—such as short-chain fatty acids—can influence beta-amyloid deposition and neuroinflammation [[18], [19], [20], [21], [22], [23]]. Moreover, bioactive compounds including probiotics, polyphenols, and omega-3 fatty acids may fortify gut barrier and BBB integrity, reducing neuroinflammatory damage [[24], [25], [26], [27], [28]]. Dietary regimens like the ketogenic and Mediterranean diets have also shown promise for neuroprotection [[29], [30], [31], [32], [33], [34]]. However, few studies have examined diet in relation to AD-specific mortality. We hypothesized that greater adherence to an anti-inflammatory diet would be associated with reduced risks of AD mortality and all-cause mortality in an aging cohort. Specifically, our present study investigates whether adherence to an anti-inflammatory diet is associated with lower AD mortality, after adjusting for key demographic, lifestyle, and clinical factors.

## 2 Methods

### 2.1 Data source

Data from the National Health and Nutrition Examination Survey (NHANES) were used for this analysis. The NHANES program began in the early 1960s and has been conducted as a series of surveys focusing on different health topics. It is a nationally representative, cross-sectional sample of a non-institutionalized U.S. population using a complex, multistage probability design [35,36]. Further information about the background, design and operation of this database is available on the NHANES website (https://www.cdc.gov/nchs/nhanes/index.html). As NHANES collects comprehensive dietary data, NHANES is an excellent database to investigate the influence of nutritional status on multiple aging-related diseases such as depression and cardiovascular disease as well as disease-specific or overall mortality to address emerging public health and clinical practice niche [[37], [38], [39], [40]].

### 2.2 Ethics statement

The study was in accordance with the ethical standards of the Helsinki Declaration of 1975, as revised in 2000. The Institutional Review Board review and approval and informed consent signed by participants were waived in the analysis of NHANES data since those data have been de-identified, and all participants in the NHANES have provided written informed consent, consistent with approval by the National Center for Health Statistics Institutional Review Board. In addition, the study design was reviewed and approved under the authority of the Institutional Review Board of Chung Shan Medical University Hospital (CSMUH No: CS2-23223).

### 2.3 Study participants

Participants ≥ 18 years old who were eligible for mortality information linkage and completed the two days dietary interview in the NHANES cycles from 2007 to 2014 were collected in the present study. Participants were excluded if their mortality status was missing or they hadn’t completed the two days dietary interview or the dietary recall status was not reliable. In the NHANES dataset, the variable “Dietary Recall Status” indicates whether dietary recall data collected using the US Department of Agriculture (USDA) Automated Multiple-Pass Method (AMPM) is considered reliable. In the present study, we included only participants who met the classification of "Reliable and met the minimum criteria," meaning they completed at least the first four of the five AMPM interview steps. Participants with outlier intake of anti-inflammatory diets were also excluded. Outliers are identified using the interquartile range (IQR) method. Specifically, an observation is classified as an outlier if it exceeds the threshold defined as the third quartile (Q3) plus 1.5 times the IQR. In this study, we first calculated the percentage of dietary intake attributed to the anti-inflammatory diet for each participant. Using SPSS, we determined that the upper boundary for detecting outliers was 29.6. Accordingly, any data points with values greater than or equal to 29.6 were considered outliers based on this standard procedure. A total of 662 participants were excluded as outliers using this approach. **Supplementary Figure S1** shows an overview of the selection process.

### 2.4 Study variables

Anti-inflammatory foods intake information was collected from the dietary interview individual food files. The examination protocol and data collection methods are fully documented in the NHANES dietary interviewer procedures manuals (https://wwwn.cdc.gov/nchs/data/nhanes/public/2013/manuals/Intrvwr_Proc_Manual.pdf). Individual intake data were collected by asking each participant to recall all the types and amounts of food that they had consumed during the 24-hour period prior to the interview (midnight to midnight). For quality assurance, all dietary interviewers were required to complete an intensive training course and were monitored throughout the data collection period. The relevant literature to define anti-inflammatory foods is listed in **Supplementary Table S1**. Briefly, specific vegetables [41], fruits [42], grains [43], starchy vegetables [44,45], beans and legumes [46,47], herbs and spices [[48], [49], [50]], proteins [51,52], and fats [53,54] were included in the anti-inflammatory foods, and the selection was based on literature review. Soup, sauce, baby food or non 100 % juice were not included in the calculations. After obtaining the amount of anti-inflammatory food consumption, we further calculated the percentage of total energy intake from anti-inflammatory foods. The percentage intake of anti-inflammatory foods was then further categorized in four groups (0 %, < 5 %, 5-9.99 %, ≥ 10 %).

### 2.5 Primary outcomes

#### 2.5.1 All-cause mortality

Mortality information was derived from public-use linked mortality files which are linked to death certificate records from the National Death Index (NDI). Each survey participant who was eligible for mortality follow-up was assigned a vital status code (Assumed alive/deceased). We used MORTSTAT to define the all-cause mortality status.

#### 2.5.2 AD mortality

The information of death from specific causes was also derived from public-use linked mortality files. The underlying cause of death was coded according to the International Statistical Classification of Diseases, Injuries, and Causes of Death, 10th Revision (ICD-10). We used UCOD_LEADING=’006’ to determine that the underlying cause of death was AD.

### 2.6 Covariates

#### 2.6.1 Demographic variables

Demographic data included age, sex, race/ethnicity, marital status, education level and poverty income ratio (a ratio of family income to poverty threshold) and were obtained from the NHANES database. Race was grouped as non-Hispanic White, non-Hispanic Black, and others. Marital status was categorized as married/living with partner, widowed/ divorced/ separated, and never married. Educational status was grouped as under 12th grade, high school graduate, and college or above. The poverty income ratio (PIR) was categorized as ≤ 1.30, 1.31-3.50, > 3.50 (richest).

#### 2.6.2 Lifestyle variables

Lifestyle factors included smoking, alcohol drinking status, body mass index (BMI), physical activity and sleep hours. Smoking status was categorized as non-smokers who reported never having smoked 100 cigarettes during their lifetime, and smokers who had smoked at least 100 cigarettes during their lifetime. Alcohol consumption was classified into heavy drinkers (who had 4/5 or more drinks every day) or not. Physical activities were obtained from physical activity questionnaire section which includes an extensive array of questions related to daily activities, leisure-time activities, and sedentary activities at home. Metabolic equivalent (MET) scores were calculated for each participant. Physical activity was classified into two groups: active (MET ≥ 600), and inactive (MET < 600) based on World Health Organization (WHO) recommendations [55,56]. BMI (weight/height^2^) was calculated during the physical examinations of the participants at the NHANES mobile examination center (MEC). According to the WHO criteria, BMI was classified into four groups: underweight (< 18.5 Kg/m^2^), normal (18.5-24.9 Kg/m^2^), overweight (25-29.9 Kg/m^2^), and obese (≥ 30.0 Kg/m^2^). Sleep hours were divided into three groups: < 7 hours, 7-9 hours, and > 9 hours, based on National Sleep Foundation recommendations [57].

#### 2.6.3 Comorbidities

Comorbid conditions comprised diabetes mellitus (DM), hypertension, cardiovascular diseases (CVD, including congestive heart failure, coronary heart disease, angina/angina pectoris, heart attack or stroke), chronic obstructive pulmonary disease (COPD, including emphysema or chronic bronchitis), and cancer, as self-reported by participants using NHANES interviewer-administered questionnaires and defined by the question “Have you ever been told by a doctor or other health professional that you had …?”. Hearing impairment was defined if the participant was wearing a hearing aid, wore a hearing aid 5 hours a week or had serious difficulty hearing. Low social contact was determined if participants self-reported having difficulty participating in social activities (such as visiting friends, attending clubs or meetings or going to parties). Depression was defined based on the Patient Health Questionnaire, a nine-item depression screening instrument, to determine the frequency of depression symptoms over the past 2 weeks. We used a score of 10 as the cut-off score to define major depression [[58], [59], [60]]. Chronic kidney disease (CKD) was defined by the urine albumin and creatinine ratio (ACR) ≥ 30 mg/g. Anemia was defined as hemoglobin < 13 g/dL in men and < 12 g/dL in women. In addition, laboratory measures of high-density lipoprotein cholesterol (HDL-C), neutrophil to lymphocyte ratio (NLR), and vitamin D in serum (25OHD2+25OHD3) were collected via blood draws as a component of the NHANES. Detailed specimen collection and processing instructions are discussed in the NHANES Laboratory/Medical Technologists Procedures Manual (LPM).

#### 2.6.4 Nutrition

Dietary data, including total protein (gm), carbohydrate (gm), dietary fiber (gm), saturated fatty acid (gm), magnesium (mg), zinc (mg), vitamin B6 (mg), vitamin B12 (mcg), vitamin C (mg), vitamin D (mcg), vitamin E (mg), niacin (mg), selenium (mcg), and calcium (mg) consumption were obtained from the dietary recall data. To reflect long-term dietary habits, we included only participants who completed two days of dietary interviews and calculated their average consumption of the above dietary nutrients. In order to control confounding, we also performed an energy adjustment. Dietary nutrition intake was adjusted to 2,000 kcal/day.

### 2.7 Statistical analysis

Data of basic characteristics are expressed as unweighted counts (weighted %) for categorical variables and as means ± standard error for continuous variables. A Chi-square test was conducted to determine differences in categorical variables, and differences in continuous variables were examined using the Complex Samples General Linear Model (CSGLM). Univariate and multivariate Cox regression were used to estimate the relative risk for all-cause mortality and for AD mortality. Variables with significance p-value < 0.05 by univariate analysis were selected and evaluated by Cox proportional hazards models. Hazard ratios (HR) and 95 % confidence intervals (CI) are depicted.

All analyses included dietary two-day sample weight (WTDR2D), stratum, and primary sampling units (PSU) per recommendations from the National Center for Health Statistics (NCHS), to perform the complex sampling design analysis to address oversampling, non-response, non-coverage, and to provide nationally representative estimates. Subgroup analyses based on gender and race were performed to explore the differences between the gender and race groups. All statistical assessments were two sided and were evaluated at the 0.05 level of significance. Statistical analyses were performed using the statistical software package SPSS complex sample module version 22.0 (IBM Corp, Armonk, NY).

### 2.8 Sensitivity analysis

To assess the robustness and specificity of our findings, we performed an additional sensitivity analysis in which the outcome was changed to mortality due to accident.

## 3 Results

### 3.1 Study population characteristics

A total of 18,795 participants were eligible for the present study of which 2,187 were deceased. Using the NHANES sample weight, the analytic sample size in the present study represented 223,577,074 non-institutionalized participants from the U.S. Basic characteristics of the study participants are shown in Table 1. Deceased participants were more likely to have consumed >10 % anti-inflammatory food (40.6 % vs. 32.5 %, p<0.001), to be older, male, non-Hispanic White, with a lower education level, a lower income level, a smoker, a heavy drinker, underweight, inactive, sleep >9 hours, comorbid with DM, hypertension, CVD, COPD, cancer, hearing impairment, depression, CKD, low social contact, and anemia (all p<0.05). Notably, unadjusted comparisons showed a higher prevalence of >10 % anti-inflammatory food intake among those who died. This likely reflects the older age of that group and possible dietary changes due to illness, factors that were accounted for in our multivariate analysis. After adjustment, the protective association of anti-inflammatory diets emerged clearly. In addition, deceased participants were more likely in an inflamed situation (NLR≥3), with lower HDL-C, and consumed less dietary nutrition mentioned in the Methods section (all p<0.05, except vitamin B12, C, D).

**Table 1:** Characteristics of participants (unweighted sample sizes and weighted %) according to mortality status (all-cause mortality).

Variable	Alive (n=16,608)	Deceased (n=2,187)	P value
**Percentage of daily caloric intake from anti-inflammatory foods (%)**			**<0.001**
0	1989 (11.5)	276 (11.2)	
< 5	4730 (29.7)	487 (24.7)	
5-9.99	4238 (26.4)	525 (23.5)	
≥ 10	5651 (32.5)	899 (40.6)	
**Demographic**			
**Age** (years)			**<0.001**
≥65	2636 (12.0)	1512 (61.7)	
45-64	5699 (36.0)	530 (29.5)	
< 45	8273 (52.0)	145 (08.8)	
**Gender**			**<0.001**
Male	7743 (47.3)	1236 (53.2)	
Female	8865 (52.7)	951 (46.8)	
**Race**			**<0.001**
Others	5944 (21.8)	360 (10.0)	
Non-Hispanic Black	3509 (11.5)	458 (11.3)	
Non-Hispanic White	7155 (66.7)	1369 (78.7)	
**Education**			**<0.001**
Under 12th grade	3597 (15.6)	761 (27.7)	
High school graduate	3531 (22.0)	575 (25.7)	
College or above	8521 (62.4)	840 (46.5)	
**Marriage**			**<0.001**
Never married	3046 (20.0)	190 (10.7)	
Widowed/Divorced/Separated	3032 (16.1)	898 (37.0)	
Married/Living with partner	9573 (64.0)	1092 (52.4)	
**Income ratio**			**<0.001**
0.00-1.30	5040 (23.4)	770 (28.9)	
>1.30-3.50	5385 (33.0)	879 (44.6)	
>3.50 (richest)	4857 (43.6)	391 (26.5)	
**Lifestyle**			
**Smoking**			**<0.001**
Smoker	6755 (42.6)	1324 (60.3)	
Non-smoker	9157 (57.4)	856 (39.7)	
**Alcohol drinking**			**<0.001**
Heavy drinker	2071 (15.2)	436 (23.8)	
Non-heavy drinker	10922 (84.8)	1312 (76.2)	
**BMI**			**0.002**
Underweight	279 (01.6)	57 (02.6)	
Overweight	5386 (32.9)	689 (31.6)	
Obese	6184 (35.5)	790 (39.8)	
Normal	4635 (30.0)	561 (26.0)	
**Physical activity**			**<0.001**
Inactive	9054 (51.5)	1702 (76.8)	
Active	7554 (48.5)	485 (23.2)	
**Sleep (hr)**			**<0.001**
< 7	6435 (36.0)	804 (35.5)	
> 9	393 (02.1)	143 (07.3)	
7-9	9754 (62.0)	1235 (57.2)	
**Comorbidities** (Yes)			
DM	1962 (08.7)	667 (27.7)	**<0.001**
Hypertension	5103 (27.3)	1423 (63.1)	**<0.001**
CVD	1170 (05.8)	754 (30.4)	**<0.001**
COPD	930 (06.0)	346 (16.1)	**<0.001**
Cancer	1224 (08.4)	541 (26.2)	**<0.001**
Hearing impairment	343 (01.8)	168 (06.9)	**<0.001**
Depression	1442 (08.3)	247 (11.3)	**0.007**
CKD	1464 (07.1)	679 (29.0)	**<0.001**
Low social contact	1228 (18.0)	598 (31.6)	**<0.001**
Anemia	1350 (06.2)	441 (18.8)	**<0.001**
**Laboratory**			
NLR ≥ 3 (1,000 cells/µL)	2287 (14.7)	649 (32.2)	**<0.001**
HDL-C < 40 (mg/dL)	3108 (19.4)	468 (24.8)	**0.001**
Vitamin D in serum (nmol/L)			0.112
< 75	10816 (62.7)	1218 (58.3)	
> 125	391 (03.5)	67 (04.4)	
75-125	4271 (33.8)	647 (37.3)	
**Dietary/nutrition** (Mean ± SE)			
Total energy (kcal/day)	2119 ± 11.63	1866 ± 25.63	**<0.001**
Protein (gm/day)	83.49 ± 0.456	72.09 ± 1.030	**<0.001**
Carbohydrate (gm/day)	256.4 ± 1.405	230.0 ± 3.174	**<0.001**
Fiber (gm/day)	17.10 ± 0.154	15.10 ± 0.308	**<0.001**
Saturated fatty acid (gm/day)	26.15 ± 0.203	23.48 ± 0.484	**<0.001**
Magnesium (mg/day)	301.9 ± 2.376	269.8 ± 4.781	**<0.001**
Zinc (mg/day)	11.72 ± 0.080	10.97 ± 0.275	**0.005**
Vitamin B6 (mg/day)	2.146 ± 0.016	1.932 ± 0.035	**<0.001**
Vitamin B12 (mcg/day)	5.284 ± 0.059	5.093 ± 0.145	0.204
Vitamin C (mg/day)	83.04 ± 1.307	81.43 ± 2.523	0.538
Vitamin D (mcg/day)	4.751 ± 0.060	4.845 ± 0.155	0.586
Vitamin E (mg/day)	9.193 ± 0.118	8.247 ± 0.271	**0.002**
Niacin (mg/day)	26.18 ± 0.148	22.65 ± 0.301	**<0.001**
Folate (mcg/day)	417.3 ± 3.334	378.1 ± 6.989	**<0.001**
Selenium (mcg/day)	115.5 ± 0.765	98.92 ± 1.261	**<0.001**
Calcium (mg/day)	982.9 ± 7.319	880.7 ± 21.45	**<0.001**

BMI: Body mass index; DM: Diabetes mellitus; CVD: Cardiovascular disease; COPD: Chronic obstructive pulmonary disease; CKD: Chronic kidney disease; NLR: Neutrophil to lymphocyte ratio; HDL-C: High-Density Lipoprotein Cholesterol.

Characteristics of participants stratified by their daily anti-inflammatory food intake percentage were described in Table 2. Participants who did not consume any anti-inflammatory foods (0 %) were more likely to be younger, male, with a lower education or income level, never married, a smoker, a heavy drinker, obese, inactive, sleep <7 hours, comorbid with depression, low social contact (all p<0.05). In addition, participants who did not consume any anti-inflammatory foods were also less comorbid with DM, hypertension, cancer, and consumed less dietary nutrition mentioned in the Methods section (all p<0.05).

**Table 2:** Characteristics of participants (unweighted sample sizes and weighted %) stratified by daily anti-inflammatory food intake percentage.

Variable	Daily anti-inflammatory food intake percentage				P value
	0 %	< 5 %	5-9.99 %	≥ 10 %	
**Status**					**<0.001**
**Alive**	1989 (91.5)	4730 (92.6)	4238 (92.1)	5651 (89.3)	
**Deceased**	276 (08.5)	487 (07.4)	525 (07.9)	899 (10.7)	
**Demographic**					
**Age** (years)					**<0.001**
65^+^	365 (10.8)	748 (10.2)	1022 (16.0)	2013 (24.1)	
45-64	616 (27.9)	1761 (35.6)	1590 (36.6)	2262 (36.9)	
<45	1284 (61.4)	2708 (54.2)	2151 (47.4)	2275 (39.1)	
**Gender**					**<0.001**
Male	1201 (54.0)	2625 (51.1)	2229 (46.2)	2924 (44.0)	
Female	1064 (46.0)	2592 (48.9)	2534 (53.8)	3626 (56.0)	
**Race**					**<0.001**
Others	707 (21.0)	1656 (19.7)	1557 (19.5)	2384 (22.7)	
Non-Hispanic Black	588 (15.1)	1074 (11.0)	989 (10.7)	1316 (11.3)	
Non-Hispanic White	970 (63.8)	2487 (69.2)	2217 (69.8)	2850 (66.1)	
**Education**					**<0.001**
Under 12th grade	674 (25.6)	1157 (17.3)	982 (13.4)	1545 (15.9)	
High school graduate	538 (26.7)	1192 (23.7)	1076 (22.4)	1300 (19.7)	
College or above	840 (47.7)	2575 (59.0)	2491 (64.2)	3455 (64.4)	
**Marriage**					**<0.001**
Never married	500 (26.9)	982 (20.1)	822 (17.5)	932 (17.0)	
Widowed/Divorced/Separated	474 (18.8)	995 (17.5)	981 (17.6)	1480 (18.3)	
Married/Living with partner	1079 (54.3)	2946 (62.4)	2748 (64.8)	3892 (64.7)	
**Income ratio**					**<0.001**
0.00-1.30	945 (34.2)	1715 (25.0)	1422 (22.3)	1728 (20.5)	
>1.30-3.50	746 (35.2)	1691 (33.0)	1608 (34.5)	2219 (34.1)	
>3.50 (richest)	400 (30.6)	1437 (41.9)	1366 (43.2)	2045 (45.4)	
**Lifestyle**					
**Smoking**					**<0.001**
Smoker	1096 (52.8)	2393 (46.7)	2036 (44.0)	2554 (39.3)	
Non-smoker	1019 (47.2)	2602 (53.3)	2566 (56.0)	3826 (60.7)	
**Alcohol drinking**					**<0.001**
Heavy drinker	390 (22.0)	813 (17.9)	594 (14.8)	710 (12.9)	
Non-heavy drinker	1354 (78.0)	3404 (82.1)	3181 (85.2)	4295 (87.1)	
**BMI**					**<0.001**
Underweight	64 (02.1)	97 (01.5)	69 (01.3)	106 (02.0)	
Overweight	640 (29.2)	1623 (31.8)	1590 (33.7)	2222 (34.1)	
Obese	889 (38.7)	2073 (39.0)	1770 (36.0)	2242 (32.0)	
Normal	639 (30.1)	1369 (27.6)	1285 (29.1)	1903 (31.9)	
**Physical activity**					**<0.001**
Inactive	1370 (60.9)	3009 (55.8)	2702 (52.2)	3675 (50.5)	
Active	895 (39.1)	2208 (44.2)	2061 (47.8)	2875 (49.5)	
**Sleep (hour)**					**<0.001**
< 7	937 (41.0)	2147 (39.5)	1842 (35.1)	2313 (31.7)	
> 9	113 (03.8)	146 (02.6)	118 (02.0)	159 (02.4)	
7-9	1210 (55.2)	2911 (57.9)	2798 (62.8)	4070 (65.9)	
**Comorbidities** (Yes)					
DM	268 (08.1)	669 (09.8)	642 (10.2)	1050 (11.7)	**0.007**
Hypertension	707 (27.8)	1683 (28.7)	1653 (31.1)	2483 (32.2)	**0.015**
CVD	230 (07.9)	449 (06.7)	465 (07.0)	780 (09.9)	**<0.001**
COPD	167 (07.1)	376 (07.4)	317 (06.0)	416 (07.2)	0.169
Cancer	152 (06.6)	419 (08.2)	435 (10.4)	759 (12.5)	**<0.001**
Hearing impairment	63 (02.3)	107 (01.6)	122 (02.5)	219 (02.6)	0.159
Depression	286 (13.3)	545 (09.9)	390 (07.7)	468 (06.4)	**<0.001**
CKD	288 (09.4)	552 (08.3)	535 (09.2)	768 (09.2)	0.411
Low social contact	271 (29.6)	481 (22.2)	415 (19.1)	659 (18.5)	**<0.001**
Anemia	233 (08.1)	444 (06.0)	418 (06.4)	696 (08.7)	**<0.001**
**Laboratory** (Mean ± SE)					
NLR ≥ 3 (1,000 cells/µL)	362 (16.7)	784 (16.5)	732 (14.9)	1058 (16.8)	0.218
HDL-C < 40 (mg/dL)	522 (23.8)	1077 (22.1)	876 (19.1)	1101 (17.3)	**<0.001**
Vitamin D in serum (nmol/L)					
< 75	1545 (69.4)	3419 (64.2)	3046 (62.2)	4024 (58.4)	**<0.001**
> 125	39 (02.7)	117 (02.8)	103 (03.4)	199 (04.8)	
75-125	473 (27.9)	1311 (33.1)	1287 (34.4)	1847 (36.8)	
**Dietary/nutrition** (Mean ± SE)					
Total energy (kcal/day)	1944 ± 26.32	2182 ± 21.17	2140 ± 19.47	2041 ± 16.09	**<0.001**
Protein (gm/day)	75.22 ± 1.377	84.31 ± 0.806	84.39 ± 0.865	81.92 ± 0.674	**<0.001**
Carbohydrate (gm/day)	238.0 ± 3.697	259.7 ± 2.852	256.9 ± 2.425	252.4 ± 1.840	**<0.001**
Fiber (gm/day)	11.91 ± 0.256	14.77 ± 0.191	17.42 ± 0.207	20.17 ± 0.228	**<0.001**
Saturated fatty acid (gm/day)	24.68 ± 0.406	27.44 ± ± 0.316	26.73 ± 0.312	24.36 ± 0.321	**<0.001**
Magnesium (mg/day)	239.7 ± 4.289	289.0 ± 3.072	307.7 ± 3.451	321.8 ± 3.502	**<0.001**
Zinc (mg/day)	10.46 ± 0.224	11.90 ± 0.135	11.85 ± 0.132	11.71 ± 0.136	**<0.001**
Vitamin B6 (mg/day)	1.772 ± 0.039	2.111 ± 0.028	2.163 ± 0.029	2.236 ± 0.023	**<0.001**
Vitamin B12 (mcg/day)	4.844 ± 0.138	5.555 ± 0.109	5.238 ± 0.099	5.184 ± 0.073	**0.001**
Vitamin C (mg/day)	53.15 ± 2.016	68.65 ± 1.885	84.20 ± 1.603	104.7 ± 1.679	**<0.001**
Vitamin D (mcg/day)	3.958 ± 0.127	4.605 ± 0.079	4.698 ± 0.110	5.219 ± 0.092	**<0.001**
Vitamin E (mg/day)	6.959 ± 0.202	8.615 ± 0.156	9.460 ± 0.199	10.01 ± 0.215	**<0.001**
Niacin (mg/day)	23.85 ± 0.390	26.94 ± 0.317	26.40 ± 0.271	25.22 ± 0.209	**<0.001**
Folate (mcg/day)	361.6 ± 7.186	405.4 ± 5.323	416.0 ± 4.894	437.7 ± 3.959	**<0.001**
Selenium (mcg/day)	106.8 ± 1.846	116.5 ± 1.234	116.1 ± 1.324	112.9 ± 1.088	**<0.001**
Calcium (mg/day)	894.0 ± 19.60	1000 ± 12.79	989.5 ± 10.99	965.7 ± 10.19	**<0.001**

BMI: Body mass index; DM: Diabetes mellitus; CVD: Cardiovascular disease; COPD: Chronic obstructive pulmonary disease; CKD: Chronic kidney disease; NLR: Neutrophil to lymphocyte ratio; HDL-C: High-Density Lipoprotein Cholesterol.

### 3.2 Factors associated with all-cause mortality

The crude and adjusted hazards for all-cause mortality from Cox proportional hazards regression analyses are presented in **Table S2**. After adjusting for significant factors in the univariate Cox proportional hazards models, percentage of daily caloric intake from anti-inflammatory foods (0 %) was a significantly increased risk of all-cause mortality [adjusted HR (aHR) =3.816, 95 % CI = 1.180-12.33]. After adjustment, the protective association of anti-inflammatory diet emerged clearly. The result of multivariate Cox regression analysis indicated that the following factors also significantly increased the risk of all-cause mortality: older age, marriage, income, sleep, CVD, COPD, cancer, CKD, low social contact, anemia, inflamed status (NLR≥3), carbohydrate intake, and Vitamin D consumption. In contrast, being of Black or other race (compared to non-Hispanic White), obese, and depression might decrease the risk of all-cause mortality (**Table S2, Supplementary** Figure 2A).

### 3.3 Factors associated with AD mortality

Table 3 depicts the crude and adjusted hazards ratios for AD mortality from Cox proportional hazards regression analyses. Due to the nature of AD mortality, we only included participants aged ≥ 45 years. After adjusting the significant factors in the univariate Cox proportional hazards models, participants who didn’t consume any anti-inflammatory food (0 %) had a higher risk of AD mortality but didn’t reach the level of statistical significance (aHR = 3.042, 95 %CI = 0.743-12.46). Older age, sleep, carbohydrate intake, and vitamin B6 consumption significantly increased the risk of AD mortality (Table 3, **Supplementary Figure 2B**).

**Table 3:** Cox proportional hazards regression analyses of AD mortality.

Variable	AD mortality	
	Crude HR (95 % CI)	Adjusted HR (95 % CI)
**Percentage of daily caloric intake from** **anti-inflammatory foods** (Ref = ≥10 %)		
0 %	2.455 (0.776-7.771)	3.042 (0.743-12.46)
< 5 %	**0.442 (0.213-0.916)**	0.784 (0.356-1.726)
5-9.9 %	0.638 (0.256-1.590)	0.942 (0.345-2.569)
**Demographic**		
**Age** (year) (Ref = 65-74 years)		
75^+^	**5.754 (2.237-14.79)**	**6.264 (2.584-15.18)**
45-64	**0.038 (0.008-0.194)**	**0.079 (0.010-0.634)**
**Gender** (Ref = Female)		
Male	1.097 (0.542-2.223)	
**Race** (Ref = Non-Hispanic White)		
Others	0.428 (0.182-1.007)	
Non-Hispanic Black	0.856 (0.265-2.766)	
**Education** (Ref = College or above)		
Under 12th grade	1.275 (0.555-2.929)	
High school graduate	0.911 (0.370-2.244)	
**Marriage** (Ref = Married)		
Widowed/Divorced/Separated	**2.486 (1.252-4.938)**	1.434 (0.768-2.679)
**Income ratio** (Ref =>3.50 (richest))		
0.00-1.30	1.819 (0.696-4.754)	
>1.30-3.50	2.078 (0.785-5.498)	
**Lifestyle**		
**Smoking** (Ref = Non-smoker)		
Smoker	0.953 (0.472-1.925)	
**Alcohol drinking** (Ref = Non-heavy drinker)		
Heavy drinker	1.455 (0.468-4.526)	
**BMI** (Ref = Normal)		
Underweight	3.694 (0.455-29.98)	
Overweight	0.923 (0.455-1.872)	
Obese	0.522 (0.242-1.128)	
**Physical activity** (Ref = Active)		
Inactive	1.894 (0.782-4.585)	
**Sleep (hour)** (Ref =7-9)		
< 7	**0.436 (0.192-0.986)**	0.536 (0.231-1.244)
> 9	**6.500 (2.379-17.75)**	**3.490 (1.312-9.281)**
**Comorbidities** (Ref =No)		
DM	1.164 (0.465-2.915)	
Hypertension	**3.629 (1.836-7.177)**	1.257 (0.574-2.753)
CVD	**4.236 (1.909-9.403)**	0.987 (0.405-2.406)
COPD	0.709 (0.211-2.386)	
Cancer	**3.062 (1.329-7.055)**	1.479 (0.636-3.441)
Hearing impairment	**3.751 (1.112-12.65)**	0.541 (0.118-2.478)
Depression	1.140 (0.374-3.468)	
CKD	**4.078 (1.782-9.332)**	1.720 (0.735-4.025)
Low social contact	**2.227 (1.023-4.849)**	1.954 (0.963-3.967)
Anemia	**3.902 (1.422-10.70)**	2.081 (0.692-6.258)
**Laboratory**		
NLR (Ref = < 3 (1,000 cells/µL))		
NLR ≥ 3	1.937 (0.870-4.313)	
HDL-C (Ref = ≥40 (mg/dL)		
HDL-C < 40	1.030 (0.356-2.974)	
Vitamin D in serum (Ref =75-125 (nmol/L))		
< 75	0.683 (0.341-1.370)	
> 125	1.048 (0.229-4.789)	
**Dietary/nutrition**^+^		
Protein (gm/day)*	0.940 (0.811-1.088)	
Carbohydrate (gm/day)*	**1.128 (1.067-1.192)**	**1.048 (0.989-1.111)**
Fiber (gm/day)*	1.039 (0.729-1.480)	
Saturated fatty acid (gm/day)*	0.690 (0.419-1.138)	
Magnesium (mg/day)*	1.003 (0.963-1.045)	
Zinc (mg/day)	1.011 (0.990-1.033)	
Vitamin B6 (mg/day)	**1.280 (1.139-1.440)**	**1.376 (1.029-1.840)**
Vitamin B12 (mcg/day)	1.015 (0.994-1.035)	
Vitamin C (mg/day)*	0.999 (0.970-1.029**)**	
Vitamin D (mcg/day)	**1.050 (1.019-1.082)**	1.007 (0.919-1.104)
Vitamin E (mg/day)	1.016 (0.978-1.056)	
Niacin (mg/day)	**1.025 (1.002-1.048)**	0.991 (0.955-1.029)
Folate (mcg/day)*	1.009 (0.996-1.021)	
Selenium (mcg/day)*	0.928 (0.857-1.005)	
Calcium (mg/day)*	**1.008 (1.003-1.014)**	0.999 (0.990-1.008)

^+^ adjusted the energy intake of each nutrition to the benchmark of 2000 Kcal.

*Increased per 10 unit

Note: only include participants aged more or equal to 45 years (n = 10,377)

AD: Alzheimer’s disease; BMI: Body mass index; DM: Diabetes mellitus; CVD: Cardiovascular disease; COPD: Chronic obstructive pulmonary disease; CKD: Chronic kidney disease; NLR: Neutrophil to lymphocyte ratio; HDL-C: High-Density Lipoprotein Cholesterol.

### 3.4 Subgroup analysis

We further performed subgroup analysis stratified by gender to explore whether there are gender differences in factors associated with AD mortality. In males, a percentage of daily caloric intake from anti-inflammatory foods (0 %) and older age had a significantly increased risk of AD mortality (Table 4, **Supplementary Table S3, Supplementary Figure 2C)**. In females, a percentage of daily caloric intake from anti-inflammatory foods was not significantly associated with AD mortality. An older age and sleep were associated with a significantly increased risk of AD mortality (Table 4, **Supplementary Table S4, Supplementary Figure 2C).**

**Table 4:** Cox proportional hazards regression analyses of AD mortality stratified by gender.

Variable	Adjusted HR (95 % CI)	
	Female	Male
**Percentage of daily caloric intake from** **anti-inflammatory foods ( %) (Ref = ≥10 %)**		
0 %	0.686 (0.168-2.803)	**12.83 (3.094-53.22)**
< 5 %	0.783 (0.314-1.951)	0.672 (0.096-4.724)
5-9.9 %	0.363 (0.105-1.252)	2.987 (0.698-12.78)
**Demographic**		
**Age** (year) (Ref = 65-74 years)		
75^+^	**3.453 (1.140-10.45)**	**15.55 (3.854-62.74)**
45-64	**0.059 (0.007-0.521)**	NA
**Race** (Ref = Non-Hispanic White)		
Others		0.300 (0.034-2.611)
Non-Hispanic Black		0.664 (0.146-3.022)
**Education** (Ref = College or above)		
Under 12th grade		
High school graduate		
**Marriage** (Ref = Married)		
Widowed/Divorced/Separated	2.107 (0.698-6.364)	
**Income ratio** (Ref =>3.50 (richest))		
0.00-1.30		
>1.30-3.50		
**Lifestyle**		
**Smoking** (Ref = Non-smoker)		
Smoker		
**Alcohol drinking** (Ref = Non-heavy drinker)		
Heavy drinker		
**BMI** (Ref = Normal)		
Underweight		
Overweight		
Obese		
**Physical activity** (Ref = Active)		
Inactive	2.103 (0.663-6.675)	
**Sleep (hour)** (Ref =7-9)		
< 7	0.622 (0.191-2.030)	0.443 (0.132-1.482)
> 9	**4.065 (1.067-15.49)**	2.636 (0.782-8.886)
**Comorbidities** (Ref =No)		
DM		
Hypertension	1.863 (0.574-6.050)	
CVD		1.483 (0.574-3.830)
COPD		
Cancer		1.727 (0.567-5.261)
Hearing impairment		
Depression		
CKD	1.299 (0.351-4.804)	1.716 (0.708-4.156)
Low social contact		2.944 (0.860-10.07)
Anemia		2.172 (0.691-6.826)
**Laboratory**		
NLR (Ref = < 3 (1,000 cells/µL))		
NLR ≥ 3		
HDL-C (Ref = ≥40 (mg/dL)		
HDL-C < 40		
Vitamin D in serum (Ref =75-125 (nmol/L))		
< 75		
> 125		
**Dietary/nutrition**^+^		
Protein (gm/day)*		0.843 (0.664-1.070)
Carbohydrate (gm/day)*	1.069 (0.972-1.175)	1.021 (0.933-1.118)
Fiber (gm/day)*		
Saturated fatty acid (gm/day)*		
Magnesium (mg/day)*		
Zinc (mg/day)		
Vitamin B6 (mg/day)	1.219 (0.810-1.834)	0.965 (0.453-2.057)
Vitamin B12 (mcg/day)		
Vitamin C (mg/day)*		
Vitamin D (mcg/day)		1.056 (0.954-1.168)
Vitamin E (mg/day)		
Niacin (mg/day)		1.084 (0.981-1.199)
Folate (mcg/day)*		0.979 (0.941-1.018)
Selenium (mcg/day)*		0.926 (0.778-1.102)
Calcium (mg/day)*		1.009 (0.995-1.022)

^+^adjusted the energy intake of each nutrition to the benchmark of 2000 Kcal.

*Increased per 10 unit.

AD: Alzheimer’s disease; BMI: Body mass index; DM: Diabetes mellitus;

CVD: Cardiovascular disease; COPD: Chronic obstructive pulmonary disease; CKD: Chronic kidney disease;

NLR: Neutrophil to lymphocyte ratio; HDL-C: High-Density Lipoprotein Cholesterol.

Table 5 depicts cox proportional hazards regression analyses of AD mortality stratified by race. Among the non-Hispanic White cohort, percentage of daily caloric intake from anti-inflammatory foods (0 %), age, sleep, and anemia were associated with a significantly increased risk of AD mortality (Table 5, **Supplementary Table S5, Supplementary Figure 2D).** In other races, those who didn’t consume any anti-inflammatory food (0 %) had a higher risk of AD mortality, but that didn’t reach statistical significance. In addition, participants with a percentage of daily caloric intake from anti-inflammatory foods 5-9.9 % vs. 10 % had a lower risk of AD mortality (aHR =0.041, 95 %CI=0.005-0.351), suggesting that even a small proportion of anti-inflammatory foods in the diet was linked to substantially lower mortality risk. Age, and marriage had a significantly increased risk of AD mortality (Table 5**, Supplementary Table S6, Supplementary Figure 2D).**

**Table 5:** Cox proportional hazards regression analyses of AD mortality stratified by race.

Variable	Adjusted HR (95 % CI)	
	White	Non-White
**Percentage of daily caloric intake from** **anti-inflammatory foods ( %) (Ref = ≥10 %)**		
0 %	**3.767 (1.041-13.63)**	2.439 (0.451-13.19)
< 5 %	0.769 (0.356-1.662)	0.933 (0.143-6.061)
5-9.9 %	1.156 (0.419-3.191)	**0.041 (0.005-0.351)**
**Demographic**		
**Age** (year) (Ref = 65-74 years)		
75^+^	**4.772 (1.747-13.03)**	**38.65 (4.355-343.1)**
45-64	**0.042 (0.005-0.346)**	NA
**Gender** (Ref = Female)		
Male		
**Education** (Ref = College or above)		
Under 12th grade		
High school graduate		
**Marriage** (Ref = Married)		
Widowed/Divorced/Separated		**10.61 (2.013-55.98)**
**Income ratio** (Ref =>3.50 (richest))		
0.00-1.30		
>1.30-3.50		
**Lifestyle**		
**Smoking** (Ref = Non-smoker)		
Smoker		
**Alcohol drinking** (Ref = Non-heavy drinker)		
Heavy drinker		
**BMI** (Ref = Normal)		
Underweight		
Overweight		
Obese		
**Physical activity** (Ref = Active)		
Inactive		
**Sleep (hour)** (Ref =7-9)		
< 7	0.573 (0.216-1.522)	
> 9	**3.825 (1.374-10.65)**	
**Comorbidities** (Ref =No)		
DM		
Hypertension	1.299 (0.588-2.870)	
CVD	0.898 (0.323-2.502)	2.736 (0.681-10.99)
COPD		
Cancer		2.626 (0.599-11.51)
Hearing impairment		
Depression		
CKD	1.927 (0.742-5.006)	
Low social contact		
Anemia	**3.439 (1.088-10.86)**	
**Laboratory**		
NLR (Ref = < 3 (1,000 cells/µL))		
NLR ≥ 3		
HDL-C (Ref = ≥40 (mg/dL)		
HDL-C < 40		
Vitamin D in serum (Ref =75-125 (nmol/L))		
< 75		
> 125		
**Dietary/nutrition**^+^		
Protein (gm/day)*		
Carbohydrate (gm/day)*	1.065 (0.996-1.139)	
Fiber (gm/day)*		
Saturated fatty acid (gm/day)*		
Magnesium (mg/day)*		1.044 (0.990-1.102)
Zinc (mg/day)		1.060 (0.985-1.141)
Vitamin B6 (mg/day)	1.230 (0.978-1.547)	1.398 (0.814-2.401)
Vitamin B12 (mcg/day)		
Vitamin C (mg/day)*		0.983 (0.921-1.050)
Vitamin D (mcg/day)	1.020 (0.899-1.156)	
Vitamin E (mg/day)		1.004 (0.963-1.046)
Niacin (mg/day)		1.001 (0.962-1.042)
Folate (mcg/day)*		
Selenium (mcg/day)*	0.930 (0.852-1.015)	
Calcium (mg/day)*	1.000 (0.988-1.011)	0.993 (0.980-1.007)

^+^ adjusted the energy intake of each nutrition to the benchmark of 2000 Kcal.

*Increased per 10 unit

AD: Alzheimer’s disease; BMI: Body mass index; DM: Diabetes mellitus; CVD: Cardiovascular disease; COPD: Chronic obstructive pulmonary disease; CKD: Chronic kidney disease; NLR: Neutrophil to lymphocyte ratio; HDL-C: High-Density Lipoprotein Cholesterol.

### 3.5 Sensitivity analysis

In the sensitivity analysis, we observed no significant association between anti-inflammatory diet intake and mortality due to accident. Compared with participants consuming ≥10 % of calories from anti-inflammatory foods, the aHR were 0.820 (95 % CI 0.224-2.997) for 0 % intake, 0.962 (95 % CI 0.364-2.544) for <5 % intake, and 2.265 (95 % CI 0.572-8.975) for 5–9.9 % intake (**Supplementary Table S7**).

## 4 Discussion

Our study showed that higher intake of anti-inflammatory foods showed only a non-significant trend toward lower AD mortality in the overall cohort. Notably, however, even modest adherence to an anti-inflammatory diet is associated with substantially lower risk of AD-related death within specific subgroups, supporting the role of chronic inflammation in AD progression and its mitigation through lifestyle. Although the narrative did not detail hazard ratios for each intake level, our analyses demonstrated a pattern of progressively lower AD mortality risk with increasing anti-inflammatory dietary intake. This stepwise decrease in risk suggests a possible dose–response relationship between greater anti-inflammatory food consumption and reduced AD mortality, though this trend is not definitive. These findings echo previous research reporting the benefits of healthy dietary patterns on cognitive function and neurodegenerative risk [[61], [62], [63]]. The observed protective effect may be mediated by reduced systemic inflammation, improved gut microbiota balance, and enhanced BBB integrity. Bioactive compounds such as polyphenols, flavonoids, and omega-3 fatty acids are likely to contribute by curbing oxidative stress and lowering pro-inflammatory cytokine levels [[64], [65], [66]].

Importantly, this apparent dose–response pattern was not uniform across all subgroups. Subgroup analyses revealed particularly strong associations among males and non-Hispanic whites. These findings imply biological sex differences such as hormonal and metabolic factors could modulate neuroinflammatory responses to diet. It’s known that dietary habits differ by sex. Men tend to consume more unhealthy foods while women tend to consume more fruits and vegetables, which could lead to different inflammation profiles. Men might experience greater risk if diet is poor, consistent with some evidence that men may be more sensitive to unhealthy diets’ cognitive effects. The finding that non-Hispanic Whites with 0 % anti-inflammatory intake had higher risk might reflect differences in baseline AD risk or diet quality across groups. Racial/ethnic differences could be tied to genetic factors or cultural dietary patterns [[67], [68], [69], [70]]. Given the especially high risk observed in males and non-Hispanic white individuals with poor diet adherence, targeted nutritional interventions or education in these subpopulations may be warranted.

These subgroup-specific findings have practical implications. The lack of a significant protective association in women and in racial/ethnic minority groups (e.g. Black or Hispanic participants) raises the question of whether a one-size-fits-all dietary recommendation is sufficient for AD risk reduction. It may be that tailored dietary strategies are needed for certain populations. For instance, women or non-White individuals might require different anti-inflammatory dietary components or additional lifestyle interventions to achieve the same neuroprotective benefit that men or White individuals appear to gain from an anti-inflammatory diet. This notion remains speculative, and we urge caution in over-interpreting these differences. Our findings do not imply that women or minorities cannot benefit from healthy diets, but rather that we did not detect a significant effect in these groups within our cohort. It is possible that other unmeasured factors (socioeconomic differences, overall diet context, or sample size limitations) contributed to the null results in those subgroups. However, we acknowledge that our analytic cohort was predominantly non-Hispanic White (over 65 % of participants), which may have influenced these subgroup findings. The elevated AD mortality risk observed in White participants with minimal anti-inflammatory food intake could simply reflect this group's larger representation and consequently greater statistical power to detect an effect, rather than indicating any true genetic or cultural predisposition to Alzheimer’s disease. If confirmed in future studies, the observed heterogeneity suggests that tailored nutritional guidance that takes sex- and culture-specific factors into account might enhance the effectiveness of dietary interventions for AD prevention. Until such data are available, public health recommendations should emphasize the general benefits of an anti-inflammatory diet while acknowledging that additional research is needed to determine how to optimize dietary advice for diverse groups.

Despite these findings, several limitations of this study must be acknowledged. First, dietary intake was assessed using self-reported 24-hour recall data, which may be prone to recall bias and measurement errors. Of the initial NHANES eligible cohort, we excluded 11,882 individuals (39 %) for incomplete or unreliable dietary recalls, yielding a final analytic sample of 18,795. This exclusion could introduce selection bias if the removed participants systematically differed. Second, residual confounding cannot be ruled out, since unmeasured factors such as genetic predisposition, medication use, cognitive status, and detailed dietary composition may influence the observed associations. Genetic risk factors, including APOE ε4 status, were not available in the NHANES dataset and thus could not be included as covariates, limiting our ability to fully control for potential confounding by genetic predisposition to Alzheimer’s disease. Third, mortality was determined using a national registry. It is important to note that the publicly available NHANES Linked Mortality Files primarily include information on the underlying cause of death, rather than multiple causes of death, which may have led to the misclassification of AD-related deaths. In the NHANES Linked Mortality Files, information on the underlying cause of death is primarily based on ICD-10 codes recorded on participants' death certificates. Specifically, Alzheimer's disease (AD) is identified as the underlying cause of death using the ICD-10 code G30. If a participant’s death certificate lists G30 as the underlying cause, NHANES classifies the death as due to Alzheimer's disease in the Linked Mortality Files. This definition aligns with the standard procedures established by the National Center for Health Statistics (NCHS) and is widely applied in epidemiological research (https://www.cdc.gov/nchs/data-linkage/mortality-public.htm).

A notable limitation of this study is that NHANES data are cross-sectional by design, capturing demographic, dietary, and health-related variables only at a single point in time[71,72]. Although linkage to mortality records provides a longitudinal dimension regarding outcomes (mortality), exposure variables such as dietary patterns and covariates were measured only once at baseline. Thus, we could not account for changes in diet or health behaviors over the follow-up period, potentially leading to exposure misclassification. This limitation should be considered when interpreting the associations observed in this analysis. These findings are best viewed as exploratory and hypothesis-generating rather than confirmatory, and they do not establish a causal relationship. Future studies should explore the mechanistic pathways underlying these associations, incorporating biomarkers of inflammation, oxidative stress, and gut microbiota diversity. Additionally, randomized controlled trials assessing the impact of structured anti-inflammatory dietary interventions, including ketogenic diets, on cognitive decline and the progression of AD are warranted to validate our findings. Given the growing burden of AD, public health strategies promoting anti-inflammatory dietary habits may hold promise for mitigating AD-related mortality and enhancing cognitive resilience in aging populations. Another limitation of our study is the wide confidence interval (CI) for the hazard ratio associated with anti-inflammatory diet intake and AD mortality (adjusted HR = 3.042, 95 % CI = 0.743–12.46). This wide CI indicates a high degree of uncertainty in the estimate, which may be attributed to the relatively small number of events in specific subgroups, such as individuals with no intake of anti-inflammatory foods. We recognize that AD deaths were extremely rare in the 45–54 year age group, so the relatively narrow confidence interval for its hazard ratio likely reflects the very small number of events and the low baseline risk in midlife. This estimate should therefore be interpreted with caution. Future studies with larger sample sizes and more detailed dietary data are necessary to provide more precise risk estimates. Moreover, potential residual confounding and measurement errors in self-reported dietary intake should be considered, as they may also contribute to variability in the results. Future longitudinal studies should track dietary patterns from midlife into older age to confirm temporal relationships, and trials should test whether dietary modifications in at-risk older adults can delay AD onset or reduce mortality. In addition, our study did not assess broader aspects of diet quality beyond the percentage of caloric intake from anti-inflammatory foods. Specifically, we did not examine participants’ overall dietary patterns (e.g., adherence to a Mediterranean or Western diet), the most commonly consumed foods, or eating behaviors such as fasting frequency. These factors can influence systemic inflammation and have been linked to mortality risk [[73], [74], [75], [76]], so their omission in our analysis is an important limitation. As a result, the absence of this information limits the interpretation of our findings and the generalizability of the results.

## 5 Conclusion

Adherence to an anti-inflammatory diet is associated with lower all-cause mortality and a reduced risk of AD-related mortality, although the latter association did not reach statistical significance in the overall cohort. Notably, significant protective effects were observed primarily in specific subgroups, particularly among male and non-Hispanic White participants who consumed at least some anti-inflammatory foods. Even a small portion of the diet (10 % of calories) being anti-inflammatory was associated with benefits, suggesting modifying dietary inflammatory content is a practical, low-cost intervention that could reduce chronic neuroinflammation and potentially reduce AD risk or mortality. Promoting an anti-inflammatory diet could be an easily implementable public health strategy to help reduce Alzheimer’s disease burden.

## Funding

This research was supported by grants from the National Science and Technology Council, Taiwan (NSTC-112-2314-B-037-037 and NSTC-113-2314-B-037-076-MY3 to S.Y.), from the Kaohsiung Medical University Hospital (KMUH112-2R61 to S.Y.), and from the Kaohsiung Medical University Gangshan Hospital (KMGH-113R010 to S.Y.). This research received funding support by the Chung Shan Medical University Hospital (CSH-2024-C-003, CSH-2025-C-005 to S.I.W.). The sponsors had no role in the design and conduct of the study; in the collection, analysis, and interpretation of data; in the preparation of the manuscript; or in the review or approval of the manuscript.

## Declaration of Generative AI and AI-assisted technologies in the writing process

We used ChatGPT o3-mini-high by OpenAI to assist with English language editing.

## CRediT authorship contribution statement

**Ching-Chi Hsu:** Writing – original draft, Visualization, Methodology, Investigation, Formal analysis, Conceptualization. **Shiow-Ing Wang:** Writing – original draft, Software, Methodology, Investigation, Funding acquisition, Formal analysis, Conceptualization. **Sebastian Yu:** Writing – original draft, Project administration, Methodology, Investigation, Funding acquisition, Formal analysis. **Eric S. Lin:** Writing – review & editing, Validation, Methodology. **James Cheng-Chung Wei:** Writing – review & editing, Supervision, Resources, Investigation, Formal analysis, Conceptualization.

## Declaration of competing interest

The authors declare that they have no known competing financial interests or personal relationships that could have appeared to influence the work reported in this paper.

## Appendix Supplementary materials

Image, application 1
