# Supplementary material for: Adherence to an anti-inflammatory diet is associated with lower Alzheimer’s disease mortality: A modifiable risk factor in a national cohort
Source: J Prev Alzheimers Dis. 2025 Jun 13;12(8):100221. doi: 10.1016/j.tjpad.2025.100221 (PMC12413709; doi:10.1016/j.tjpad.2025.100221)
Supplement: Supplementary file 1 [file mmc1.docx]

**Table S1.** List of anti-inflammatory foods used in the present study

| **Vegetables**   - Artichoke - Arugula - Asparagus - Bamboo shoots - Beets - Bok choy - Broccoli - Brussels sprouts - Cabbages - Carrots - Cauliflower - Celery - Chard - Cucumber - Eggplant - Fennel - Garlic - Lettuce - Hearts of palm - Kale - Leeks - Mushrooms - Onion - Peppers - Radishes - Rhubarb - Shallots - Spinach - Tomato - Turnips - Watercress - Zucchini | **Proteins**   - Edamame - Tempeh - Tofu - Soy milk - Soy nuts - Bluefin tuna - Clams - Crab - Flounder/ sole - Grouper - Halibut - Herring - Lobster - Makerel - Mussels - Oysters - Rainbow trout - Salmon - Sardines - Shrimp - Snapper - Striped bass - Tuna | **Fruits**   - Apple - Apricot - Banana - Blackberries - Blueberries - Cantaloupe - Cherries - Clementine - Dates - Figs - Grapes - Guava - Honeydew - Kiwi - Lemon - Mongo - Nectarine - Orange - Papaya - Pear pineapple - Plum - Pomegranate - Starfruit - Strawberries - Watermelon |
| --- | --- | --- |
| **Grains**   - Amaranth - Barley - Black rice - Brown rice - Buckwheat - Kasha - Bulgur - Farro - Millet - Oats - Quinoa - Wild rice | **Starchy vegetables**   - Squash (all types) - Potatoes (all types) - Jerusalem artichoke - Jicama - Parsnips - Pumpkin - Yams | **Beans and legumes**   - Beans (all types) - String beans - Peas - Soybeans - Lentils |
| **Fats**   - Almonds - Avocado - Brazil nuts - Cashews - Canola oil - Chia seeds - Flax seeds - Hazelnuts - Hemp seeds - Macadamia - Olives - Peanuts - Pecans - Pistachios - Pumpkin seeds - Sesame seeds - Sunflower seeds - Walnuts | **Herbs and spices**   - Basil - Pepper - Cilantro - Cinnamon - Cloves - Cumin - Curry - Dill - Ginger - Garlic - Mint - Oregano - Parsley - Rosemary - Sage - Thyme - Turmeric |  |

**Table S2.** Cox proportional hazards regression analyses of all-cause mortality

| **Variable** | | **All-cause mortality** | |
| --- | --- | --- | --- |
|  |  | Crude HR  (95% CI) | Adjusted HR  (95% CI) |
| **Percentage of daily caloric intake from anti-inflammatory foods** (%) (Ref = ≥10%) | | |  |
|  | 0 % | **0.786 (0.619-0.999)** | **3.816 (1.180-12.33)** |
|  | < 5% | **0.682 (0.554-0.838)** | 0.697 (0.212-2.296) |
|  | 5-9.9% | **0.726 (0.592-0.891)** | 0.412 (0.074-2.285) |
| **Demographic** | |  |  |
|  | **Age** (yr) (Ref = <45 yr) |  |  |
|  | 65+ | **25.60 (20.03-32.72)** | **13.88 (5.704-33.77)** |
|  | 45-64 | **4.744 (3.640-6.182)** | **6.382 (2.515-16.19)** |
|  | **Gender** (Ref = Female) |  |  |
|  | Male | **1.267 (1.120-1.433)** | 1.211 (0.903-1.623) |
|  | **Race** (Ref = Non-Hispanic White) |  |  |
|  | Others | **0.421 (0.349-0.508)** | **0.560 (0.409-0.768)** |
|  | Non-Hispanic Black | **0.852 (0.738-0.988)** | **0.747 (0.574-0.972)** |
|  | **Education** (Ref = College or above) |  |  |
|  | Under 12th grade | **2.158 (1.783-2.611)** | 1.101 (0.885-1.371) |
|  | High school graduate | **1.483 (1.256-1.752)** | 1.033 (0.846-1.260) |
|  | **Marriage** (Ref = Married) |  |  |
|  | Never married | **0.659 (0.509-0.855)** | **1.655 (1.132-2.421)** |
|  | Widowed/Divorced/Separated | **2.649 (2.337-3.004)** | **1.667 (1.320-2.106)** |
|  | **Income ratio** (Ref =>3.50 (richest)) |  |  |
|  | 0.00-1.30 | **2.029 (1.648-2.498)** | **1.443 (1.045-1.992)** |
|  | >1.30-3.50 | **2.165 (1.764-2.658)** | **1.437 (1.112-1.856)** |
| **Lifestyle** | |  |  |
|  | **Smoking** (Ref = Non-smoker) |  |  |
|  | Smoker | **1.926 (1.640-2.262)** | 1.188 (0.974-1.449) |
|  | **Alcohol drinking** (Ref = Non-heavy drinker) |  |  |
|  | Heavy drinker | **1.688 (1.391-2.050)** | 1.041 (0.798-1.358) |
|  | **BMI** (Ref = Normal) |  |  |
|  | Underweight | **1.828 (1.233-2.710)** | 1.095 (0.584-2.052) |
|  | Overweight | 1.112 (0.942-1.312) | 0.807 (0.646-1.010) |
|  | Obese | **1.296 (1.092-1.538)** | **0.765 (0.614-0.955)** |
|  | **Physical activity** (Ref = Active) |  |  |
|  | Inactive | **2.958 (2.633-3.323)** | **1.613 (1.339-1.943)** |
|  | **Sleep (hr)** (Ref =7-9) |  |  |
|  | < 7 | 1.067 (0.929-1.224) | 0.955 (0.792-1.153) |
|  | > 9 | **3.623 (2.743-4.784)** | **1.440 (1.048-1.978)** |
| **Comorbidities** (Ref =No) | |  |  |
|  | DM | **3.778 (3.312-4.310)** | 1.083 (0.825-1.420) |
|  | Hypertension | **4.436 (3.828-4.934)** | **1.352 (1.098-1.665)** |
|  | CVD | **6.061 (5.302-6.927)** | **1.330 (1.120-1.578)** |
|  | COPD | **2.735 (2.292-3.263)** | **1.365 (1.123-1.659)** |
|  | Cancer | **3.594 (3.094-4.175)** | **1.466 (1.212-1.773)** |
|  | Hearing impairment | **4.674 (3.763-5.804)** | 1.085 (0.749-1.572) |
|  | Depression | **1.382 (1.099-1.737)** | **0.675 (0.520-0.877)** |
|  | CKD | **4.770 (4.096-5.555)** | **2.107 (1.734-2.560)** |
|  | Low social contact | **1.942 (1.681-2.244)** | **1.831 (1.473-2.275)** |
|  | Anemia | **3.344 (2.802-3.991)** | **1.797 (1.406-2.295)** |
| **Laboratory** | |  |  |
|  | NLR (Ref = < 3 (1,000 cells/µL)) |  |  |
|  | NLR ≥ 3 | **2.694 (2.353-3.085)** | **1.491 (1.253-1.774)** |
|  | HDL-C (Ref = ≥40 (mg/dL) |  |  |
|  | HDL-C < 40 | **1.336 (1.126-1.585)** | 1.119 (0.889-1.408) |
|  | Vitamin D in serum (Ref =75-125 (nmol/L)) |  |  |
|  | < 75 | 0.852 (0.718-1.011) |  |
|  | > 125 | 1.218 (0.802-1.851) |  |
| **Dietary/nutrition^+^** | |  |  |
|  | Protein (gm/day)^*^ | 0.983 (0.948-1.019) |  |
|  | Carbohydrate (gm/day)^*^ | **1.020 (1.008-1.031)** | **1.033 (1.008-1.059)** |
|  | Fiber (gm/day)^*^ | 1.051 (0.966-1.143) |  |
|  | Saturated fatty acid (gm/day)^*^ | 1.067 (0.970-1.173) |  |
|  | Magnesium (mg/day)^*^ | 1.005 (0.998-1.013) |  |
|  | Zinc (mg/day) | **1.014 (1.007-1.020)** | 0.991 (0.975-1.007) |
|  | Vitamin B6 (mg/day) | **1.048 (1.005-1.094)** | 0.997 (0.893-1.113) |
|  | Vitamin B12 (mcg/day) | **1.015 (1.008-1.023)** | 0.994 (0.977-1.011) |
|  | Vitamin C (mg/day)^*^ | **1.015 (1.008-1.021)** | 1.009 (0.997-1.021) |
|  | Vitamin D (mcg/day) | **1.037 (1.023-1.052)** | **1.021 (1.000-1.043)** |
|  | Vitamin E (mg/day) | 1.007 (0.997-1.017) |  |
|  | Niacin (mg/day) | 0.998 (0.991-1.005) |  |
|  | Folate (mcg/day)^*^ | **1.004 (1.000-1.007)** | 0.997 (0.991-1.003) |
|  | Selenium (mcg/day)^*^ | 0.988 (0.969-1.008) |  |
|  | Calcium (mg/day)^*^ | 1.001 (0.999-1.003) |  |
| Interaction term | |  | **p value** |
|  | Anti-inflammatory diet * age |  | **0.010** |
|  | Anti-inflammatory diet * gender |  | **0.027** |

+ adjusted the energy intake of each nutrition to the benchmark of 2000 Kcal.

*Increased per 10 unit

BMI: Body mass index

DM: Diabetes mellitus

CVD: Cardiovascular disease

COPD: Chronic obstructive pulmonary disease

CKD: Chronic kidney disease

NLR: Neutrophil to lymphocyte ratio

HDL-C: High-Density Lipoprotein Cholesterol

**Table S3.** Cox proportional hazards regression analyses of AD mortality_Male

| Variables | | AD mortality | |
| --- | --- | --- | --- |
|  |  | Crude HR  (95% CI) | Adjusted HR  (95% CI) |
| **Anti-inflammatory diet** (%) (Ref = ≧10) | |  |  |
|  | 0 | **8.020 (2.109-30.49)** | **12.83 (3.094-53.22)** |
|  | < 5% | 0.388 (0.100-1.512) | 0.672 (0.096-4.724) |
|  | 5-9.9% | 1.735 (0.459-6.554) | 2.987 (0.698-12.78) |
| **Demographic** | |  |  |
|  | **Age** (years) (Ref = 65-74 y) |  |  |
|  | 75+ |  | **15.55 (3.854-62.74)** |
|  | 45-64 |  | NA |
|  | **Race** (Ref = Non-Hispanic White) |  |  |
|  | Others | **0.335 (0.112-0.998)** | 0.300 (0.034-2.611) |
|  | Non-Hispanic Black | 0.399 (0.063-2.533) | 0.664 (0.146-3.022) |
|  | **Education** (Ref = College or above) |  |  |
|  | Under 12th grade | 0.624 (0.218-1.785) |  |
|  | High school graduate | 1.293 (0.416-4.012) |  |
|  | **Marriage** (Ref = Married) |  |  |
|  | Widowed/Divorced/Separated | 1.212 (0.390-3.766) |  |
|  | **Income ratio** (Ref =>3.50 (richest)) |  |  |
|  | 0.00-1.30 | 1.115 (0.189-6.589) |  |
|  | >1.30-3.50 | 2.108 (0.516-8.605) |  |
| **Lifestyle** | |  |  |
|  | **Smoking** (Ref = Non-smoker) |  |  |
|  | Smoker | 1.855 (0.542-6.351) |  |
|  | **Alcohol drinking** (Ref = Non-heavy drinker) |  |  |
|  | Heavy drinker | 1.995 (0.591-6.731) |  |
|  | **BMI** (Ref = Normal) |  |  |
|  | Underweight | NA |  |
|  | Overweight | 0.968 (0.316-2.962) |  |
|  | Obese | 0.417 (0.131-1.325) |  |
|  | **Physical activity** (Ref = Active) |  |  |
|  | Inactive | 1.313 (0.416-4.145) |  |
|  | **Sleep (hours)** (Ref =7-9) |  |  |
|  | < 7 | 0.308 (0.094-1.003) | 0.443 (0.132-1.482) |
|  | > 9 | **4.380 (1.146-16.74)** | 2.636 (0.782-8.886) |
| **Comorbidities** (Ref =No) | |  |  |
|  | DM | 0.344 (0.095-1.242) |  |
|  | Hypertension | 2.654 (0.933-7.554) |  |
|  | CVD | **8.583 (3.509-20.99)** | 1.483 (0.574-3.830) |
|  | COPD | 1.186 (0.211-6.675) |  |
|  | Cancer | **4.360 (1.477-12.87)** | 1.727 (0.567-5.261) |
|  | Hearing impairment | 2.471 (0.671-9.093) |  |
|  | Depression | 2.416 (0.583-10.00) |  |
|  | CKD | **5.220 (1.600-17.03)** | 1.716 (0.708-4.156) |
|  | Low social contact | **3.552 (1.032-12.22)** | 2.944 (0.860-10.07) |
|  | Anemia | **6.702 (2.043-21.98)** | 2.172 (0.691-6.826) |
| **Laboratory** | |  |  |
|  | NLR (Ref = < 3 (1000 cell/uL)) |  |  |
|  | NLR ≧ 3 | 2.027 (0.634-6.488) |  |
|  | HDL-C (Ref = ≧40 (mg/dL) |  |  |
|  | HDL-C < 40 | 1.357 (0.401-4.589) |  |
|  | Vitamin D in serum (Ref =75-125 (nmol/L)) |  |  |
|  | < 75 | 0.447 (0.148-1.347) |  |
|  | > 125 | 1.500 (0.155-14.53) |  |
| **Dietary/nutrition^+^** | |  |  |
|  | Protein (gm/day)^*^ | **0.831 (0.696-0.992)** | 0.843 (0.664-1.070) |
|  | Carbohydrate (gm/day)^*^ | **1.152 (1.049-1.265)** | 1.021 (0.933-1.118) |
|  | Fiber (gm/day)^*^ | 0.949 (0.468-1.923) |  |
|  | Saturated fatty acid (gm/day)^*^ | 0.558 (0.283-1.101) |  |
|  | Magnesium (mg/day)^*^ | 0.996 (0.990-1.002) |  |
|  | Zinc (mg/day) | 1.017 (0.986-1.049) |  |
|  | Vitamin B6 (mg/day) | **1.314 (1.070-1.613)** | 0.965 (0.453-2.057) |
|  | Vitamin B12(mcg/day) | 1.013 (0.989-1.037) |  |
|  | Vitamin C (mg/day)^*^ | 0.997 (0.938-1.059) |  |
|  | Vitamin D (mcg/day) | **1.053 (1.013-1.096)** | 1.056 (0.954-1.168) |
|  | Vitamin E (mg/day) | 1.038 (0.993-1.085) |  |
|  | Niacin (mg/day) | **1.041 (1.019-1.064)** | 1.084 (0.981-1.199) |
|  | Folate (mcg/day)^*^ | **1.014 (1.000-1.027**) | 0.979 (0.941-1.018) |
|  | Selenium (mcg/day)^*^ | **0.875 (0.781-0.979**) | 0.926 (0.778-1.102) |
|  | Calcium (mg/day)^*^ | **1.013 (1.005-1.021)** | 1.009 (0.995-1.022) |

Note: only include subjects aged more or equal to 45y

+ adjusted the energy intake of each nutrition to the benchmark of 2000 Kcal.

*Increased per 10 unit

AD: Alzheimer’s disease

BMI: Body mass index,

DM: Diabetes mellitus

CVD: Cardiovascular disease

COPD: Chronic obstructive pulmonary disease

CKD: Chronic kidney disease

NLR: Neutrophil to lymphocyte ratio

HDL-C: High-Density Lipoprotein Cholesterol

**Table S4.** Cox proportional hazards regression analyses of AD mortality_Female

| Variables | | AD mortality | |
| --- | --- | --- | --- |
|  |  | Crude HR  (95% CI) | Adjusted HR  (95% CI) |
| **Anti-inflammatory diet** (%) (Ref = ≧10) | |  |  |
|  | 0 | 0.416 (0.097-1.791) | 0.686 (0.168-2.803) |
|  | < 5% | 0.498 (0.194-1.284) | 0.783 (0.314-1.951) |
|  | 5-9.9% | **0.274 (0.083-0.904)** | 0.363 (0.105-1.252) |
| **Demographic** | |  |  |
|  | **Age** (years) (Ref = 65-74 y) |  |  |
|  | 75+ | **5.497 (1.888-16.00)** | **3.453 (1.140-10.45)** |
|  | 45-64 | **0.041 (0.004-0.387)** | **0.059 (0.007-0.521)** |
|  | **Race** (Ref = Non-Hispanic White) |  |  |
|  | Others | 0.526 (0.141-1.970) |  |
|  | Non-Hispanic Black | 1.296 (0.305-5.507) |  |
|  | **Education** (Ref = College or above) |  |  |
|  | Under 12th grade | 1.858 (0.711-4.855) |  |
|  | High school graduate | 0.566 (0.180-1.783) |  |
|  | **Marriage** (Ref = Married) |  |  |
|  | Widowed/Divorced/Separated | **4.899 (1.568-15.31)** | 2.107 (0.698-6.364) |
|  | **Income ratio** (Ref =>3.50 (richest)) |  |  |
|  | 0.00-1.30 | 2.315 (0.723-7.410) |  |
|  | >1.30-3.50 | 2.040 (0.627-6.632) |  |
| **Lifestyle** | |  |  |
|  | **Smoking** (Ref = Non-smoker) |  |  |
|  | Smoker | 0.493 (0.170-1.429) |  |
|  | **Alcohol drinking** (Ref = Non-heavy drinker) |  |  |
|  | Heavy drinker | 0.647 (0.069-6.019) |  |
|  | **BMI** (Ref = Normal) |  |  |
|  | Underweight | 0.812 (0.333-1.978) |  |
|  | Overweight | 0.614 (0.222-1.701) |  |
|  | Obese |  |  |
|  | **Physical activity** (Ref = Active) |  |  |
|  | Inactive | **3.184 (1.009-10.04)** | 2.103 (0.663-6.675) |
|  | **Sleep (hours)** (Ref =7-9) |  |  |
|  | < 7 | 0.581 (0.187-1.802) | 0.622 (0.191-2.030) |
|  | > 9 | **8.731 (2.452-31.08)** | **4.065 (1.067-15.49)** |
| **Comorbidities** (Ref =No) | |  |  |
|  | DM | 2.281 (0.676-7.692) |  |
|  | Hypertension | **5.070 (1.885-13.64)** | 1.863 (0.574-6.050) |
|  | CVD | 1.698 (0.572-5.038) |  |
|  | COPD | 0.419 (0.083-2.111) |  |
|  | Cancer | 2.127 (0.665-6.807) |  |
|  | Hearing impairment | 5.260 (0.932-29.68) |  |
|  | Depression | 0.574 (0.127-2.596) |  |
|  | CKD | **3.326 (1.107-9.994)** | 1.299 (0.351-4.804) |
|  | Low social contact | 1.407 (0.545-3.635) |  |
|  | Anemia | 2.295 (0.457-11.52) |  |
| **Laboratory** | |  |  |
|  | NLR (Ref = < 3 (1000 cell/uL)) |  |  |
|  | NLR ≧ 3 | 1.765 (0.652-4.777) |  |
|  | HDL-C (Ref = ≧40 (mg/dL) |  |  |
|  | HDL-C < 40 | 0.261 (0.032-2.155) |  |
|  | Vitamin D in serum (Ref =75-125 (nmol/L)) |  |  |
|  | < 75 | 1.004 (0.408-2.471) |  |
|  | > 125 | 1.006 (0.130-7.772) |  |
| **Dietary/nutrition^+^** | |  |  |
|  | Protein (gm/day)^*^ | 1.021 (0.829-1.256) |  |
|  | Carbohydrate (gm/day)^*^ | **1.108 (1.034-1.188)** | 1.069 (0.972-1.175) |
|  | Fiber (gm/day)^*^ | 1.121 (0.852-1.475) |  |
|  | Saturated fatty acid (gm/day)^*^ | 0.831 (0.393-1.756) |  |
|  | Magnesium (mg/day)^*^ | 1.012 (0.976-1.049) |  |
|  | Zinc (mg/day) | 1.006 (0.971-1.043) |  |
|  | Vitamin B6 (mg/day) | **1.265 (1.085-1.474)** | 1.219 (0.810-1.834) |
|  | Vitamin B12(mcg/day) | 1.018 (0.967-1.071) |  |
|  | Vitamin C (mg/day)^*^ | 1.001 (0.978-1.025) |  |
|  | Vitamin D (mcg/day) | 1.046 (0.989-1.106) |  |
|  | Vitamin E (mg/day) | 0.987 (0.921-1.059) |  |
|  | Niacin (mg/day) | 1.006 (0.966-1.047) |  |
|  | Folate (mcg/day)^*^ | 1.003 (0.980-1.027) |  |
|  | Selenium (mcg/day)^*^ | 0.968 (0.872-1.074) |  |
|  | Calcium (mg/day)^*^ | 1.005 (0.996-1.014) |  |

Note: only include subjects aged more or equal to 45y

+ adjusted the energy intake of each nutrition to the benchmark of 2000 Kcal.

*Increased per 10 unit

AD: Alzheimer’s disease

BMI: Body mass index,

DM: Diabetes mellitus

CVD: Cardiovascular disease

COPD: Chronic obstructive pulmonary disease

CKD: Chronic kidney disease

NLR: Neutrophil to lymphocyte ratio

HDL-C: High-Density Lipoprotein Cholesterol

**Table S5.** Cox proportional hazards regression analyses of AD mortality_White

| Variables | | AD mortality | |
| --- | --- | --- | --- |
|  |  | Crude HR  (95% CI) | Adjusted HR  (95% CI) |
| **Anti-inflammatory diet** (%) (Ref = ≧10) | |  |  |
|  | 0 | 2.952 (0.813-10.71) | **3.767 (1.041-13.63)** |
|  | < 5% | **0.366 (0.167-0.803)** | 0.769 (0.356-1.662) |
|  | 5-9.9% | 0.763 (0.302-1.927) | 1.156 (0.419-3.191) |
| **Demographic** | |  |  |
|  | **Age** (years) (Ref = 65-74 y) |  |  |
|  | 75+ | **4.327 (1.620-11.55)** | **4.772 (1.747-13.03)** |
|  | 45-64 | **0.043 (0.008-0.222)** | **0.042 (0.005-0.346)** |
|  | **Sex** (Ref = Female) |  |  |
|  | Male | 1.243 (0.578-2.673) |  |
|  | **Education** (Ref = College or above) |  |  |
|  | Under 12th grade | 1.588 (0.561-4.498) |  |
|  | High school graduate | 0.947 (0.342-2.618) |  |
|  | **Marriage** (Ref = Married) |  |  |
|  | Widowed/Divorced/Separated | 1.912 (0.916-3.989) |  |
|  | **Income ratio** (Ref =>3.50 (richest)) |  |  |
|  | 0.00-1.30 | 2.193 (0.693-6.943) |  |
|  | >1.30-3.50 | 2.803 (0.984-7.984) |  |
| **Lifestyle** | |  |  |
|  | **Smoking** (Ref = Non-smoker) |  |  |
|  | Smoker | 0.935 (0.441-1.984) |  |
|  | **Alcohol drinking** (Ref = Non-heavy drinker) |  |  |
|  | Heavy drinker | 2.033 (0.613-6.736) |  |
|  | **BMI** (Ref = Normal) |  |  |
|  | Underweight | NA |  |
|  | Overweight | 0.901 (0.419-1.936) |  |
|  | Obese | 0.518 (0.219-1.225) |  |
|  | **Physical activity** (Ref = Active) |  |  |
|  | Inactive | 1.735 (0.652-4.618) |  |
|  | **Sleep (hours)** (Ref =7-9) |  |  |
|  | < 7 | 0.433 (0.170-1.101) | 0.573 (0.216-1.522) |
|  | > 9 | **6.661 (2.112-21.01)** | **3.825 (1.374-10.65)** |
| **Comorbidities** (Ref =No) | |  |  |
|  | DM | 0.831 (0.196-3.528) |  |
|  | Hypertension | **3.390 (1.652-6.958)** | 1.299 (0.588-2.870) |
|  | CVD | **4.006 (1.632-9.831)** | 0.898 (0.323-2.502) |
|  | COPD | 0.784 (0.229-2.685) |  |
|  | Cancer | 2.355 (0.929-5.972) |  |
|  | Hearing impairment | 3.274 (0.869-12.33) |  |
|  | Depression | 1.323 (0.371-4.723) |  |
|  | CKD | **4.776 (1.802-12.65)** | 1.927 (0.742-5.006) |
|  | Low social contact | 2.278 (0.898-5.776) |  |
|  | Anemia | **5.605 (1.825-17.21)** | **3.439 (1.088-10.86)** |
| **Laboratory** | |  |  |
|  | NLR (Ref = < 3 (1000 cell/uL)) |  |  |
|  | NLR ≧ 3 | 2.045 (0.863-4.848) |  |
|  | HDL-C (Ref = ≧40 (mg/dL) |  |  |
|  | HDL-C < 40 | 0.937 (0.252-3.482) |  |
|  | Vitamin D in serum (Ref =75-125 (nmol/L)) |  |  |
|  | < 75 | 0.893 (0.420-1.900) |  |
|  | > 125 | 1.219 (0.261-5.692) |  |
| **Dietary/nutrition^+^** | |  |  |
|  | Protein (gm/day)^*^ | 0.894 (0.755-1.059) |  |
|  | Carbohydrate (gm/day)^*^ | **1.156 (1.088-1.228)** | 1.065 (0.996-1.139) |
|  | Fiber (gm/day)^*^ | 0.971 (0.631-1.496) |  |
|  | Saturated fatty acid (gm/day)^*^ | 0.736 (0.435-1.246) |  |
|  | Magnesium (mg/day)^*^ | 0.987 (0.940-1.036) |  |
|  | Zinc (mg/day) | 1.001 (0.946-1.060) |  |
|  | Vitamin B6 (mg/day) | **1.250 (1.094-1.429)** | 1.230 (0.978-1.547) |
|  | Vitamin B12(mcg/day) | 1.014 (0.992-1.037) |  |
|  | Vitamin C (mg/day)^*^ | 0.993 (0.956-1.032) |  |
|  | Vitamin D (mcg/day) | **1.054 (1.011-1.099)** | 1.020 (0.899-1.156) |
|  | Vitamin E (mg/day) | 1.005 (0.945-1.068) |  |
|  | Niacin (mg/day) | 1.018 (0.992-1.046) |  |
|  | Folate (mcg/day)^*^ | 1.008 (0.994-1.021) |  |
|  | Selenium (mcg/day)^*^ | **0.907 (0.829-0.992)** | 0.930 (0.852-1.015) |
|  | Calcium (mg/day)^*^ | **1.008 (1.001-1.014)** | 1.000 (0.988-1.011) |

Note: only include subjects aged more or equal to 45y

+ adjusted the energy intake of each nutrition to the benchmark of 2000 Kcal.

*Increased per 10 unit

AD: Alzheimer’s disease

BMI: Body mass index,

DM: Diabetes mellitus

CVD: Cardiovascular disease

COPD: Chronic obstructive pulmonary disease

CKD: Chronic kidney disease

NLR: Neutrophil to lymphocyte ratio

HDL-C: High-Density Lipoprotein Cholesterol

**Table S6.** Cox proportional hazards regression analyses of AD mortality_non-White

| Variables | | AD mortality | |
| --- | --- | --- | --- |
|  |  | Crude HR  (95% CI) | Adjusted HR  (95% CI) |
| **Anti-inflammatory diet** (%) (Ref = ≧10) | |  |  |
|  | 0 | 1.192 (0.178-7.998) | 2.439 (0.451-13.19) |
|  | < 5% | 0.753 (0.163-3.489) | 0.933 (0.143-6.061) |
|  | 5-9.9% | **0.037 (0.004-0.382)** | **0.041 (0.005-0.351)** |
| **Demographic** | |  |  |
|  | **Age** (years) (Ref = 65-74 y) |  |  |
|  | 75+ | **62.66 (6.726-583.8)** | **38.65 (4.355-343.1)** |
|  | 45-64 | NA | NA |
|  | **Sex** (Ref = Female) |  |  |
|  | Male | 0.537 (0.158-1.831) |  |
|  | **Education** (Ref = College or above) |  |  |
|  | Under 12th grade | 1.100 (0.220-5.496) |  |
|  | High school graduate | 0.794 (0.112-5.630) |  |
|  | **Marriage** (Ref = Married) |  |  |
|  | Widowed/Divorced/Separated | **23.49 (4.064-135.8)** | **10.61 (2.013-55.98)** |
|  | **Income ratio** (Ref =>3.50 (richest)) |  |  |
|  | 0.00-1.30 | 1.151 (0.160-8.287) |  |
|  | >1.30-3.50 | 0.386 (0.055-2.708) |  |
| **Lifestyle** | |  |  |
|  | **Smoking** (Ref = Non-smoker) |  |  |
|  | Smoker | 0.962 (0.198-4.660) |  |
|  | **Alcohol drinking** (Ref = Non-heavy drinker) |  |  |
|  | Heavy drinker | NA |  |
|  | **BMI** (Ref = Normal) |  |  |
|  | Underweight | NA |  |
|  | Overweight | 1.136 (0.236-5.468) |  |
|  | Obese | 0.750 (0.176-3.194) |  |
|  | **Physical activity** (Ref = Active) |  |  |
|  | Inactive | 4.036 (0.467-34.83) |  |
|  | **Sleep (hours)** (Ref =7-9) |  |  |
|  | < 7 | 0.559 (0.105-2.966) |  |
|  | > 9 | 7.474 (0.917-60.95) |  |
| **Comorbidities** (Ref =No) | |  |  |
|  | DM | 3.460 (0.849-14.10) |  |
|  | Hypertension | 6.434 (0.900-46.00) |  |
|  | CVD | **5.453 (1.132-26.25)** | 2.736 (0.681-10.99) |
|  | COPD | NA |  |
|  | Cancer | **10.05 (2.143-47.14)** | 2.626 (0.599-11.51) |
|  | Hearing impairment | 6.931 (0.673-71.37) |  |
|  | Depression | 0.725 (0.080-6.538) |  |
|  | CKD | 2.646 (0.522-13.40) |  |
|  | Low social contact | 2.778 (0.636-12.12) |  |
|  | Anemia | 1.291 (0.321-5.192) |  |
| **Laboratory** | |  |  |
|  | NLR (Ref = < 3 (1000 cell/uL)) |  |  |
|  | NLR ≧ 3 | 0.999 (0.177-5.639) |  |
|  | HDL-C (Ref = ≧40 (mg/dL) |  |  |
|  | HDL-C < 40 | 1.555 (0.422-5.734) |  |
|  | Vitamin D in serum (Ref =75-125 (nmol/L)) |  |  |
|  | < 75 | 0.247 (0.056-1.097) |  |
|  | > 125 | NA |  |
| **Dietary/nutrition^+^** | |  |  |
|  | Protein (gm/day)^*^ | 1.146 (0.937-1.402) |  |
|  | Carbohydrate (gm/day)^*^ | 1.046 (0.915-1.196) |  |
|  | Fiber (gm/day)^*^ | 1.357 (0.856-2.153) |  |
|  | Saturated fatty acid (gm/day)^*^ | 0.307 (0.085-1.111) |  |
|  | Magnesium (mg/day)^*^ | **1.057 (1.022-1.094**) | 1.044 (0.990-1.102) |
|  | Zinc (mg/day) | **1.076 (1.028-1.127)** | 1.060 (0.985-1.141) |
|  | Vitamin B6 (mg/day) | **1.405 (1.160-1.701)** | 1.398 (0.814-2.401) |
|  | Vitamin B12(mcg/day) | 1.012 (0.975-1.051) |  |
|  | Vitamin C (mg/day)^*^ | **1.027 (1.000-1.054)** | 0.983 (0.921-1.050) |
|  | Vitamin D (mcg/day) | 1.043 (1.000-1.088) |  |
|  | Vitamin E (mg/day) | **1.046 (1.026-1.068)** | 1.004 (0.963-1.046) |
|  | Niacin (mg/day) | **1.046 (1.019-1.073)** | 1.001 (0.962-1.042) |
|  | Folate (mcg/day)^*^ | 1.013 (0.995-1.032) |  |
|  | Selenium (mcg/day)^*^ | 1.021 (0.933-1.117) |  |
|  | Calcium (mg/day)^*^ | **1.010 (1.005-1.015)** | 0.993 (0.980-1.007) |

+ adjusted the energy intake of each nutrition to the benchmark of 2000 Kcal.

*Increased per 10 unit

AD: Alzheimer’s disease

BMI: Body mass index,

DM: Diabetes mellitus

CVD: Cardiovascular disease

COPD: Chronic obstructive pulmonary disease

CKD: Chronic kidney disease

NLR: Neutrophil to lymphocyte ratio

HDL-C: High-Density Lipoprotein Cholesterol

**Table S7.** Cox proportional hazards regression analyses_ mortality due to accident

| Variables | | Mortality due to accident | |
| --- | --- | --- | --- |
|  |  | Crude HR  (95% CI) | Adjusted HR  (95% CI) |
| **Anti-inflammatory diet** (%) (Ref = ≧10) | |  |  |
|  | 0 | 2.200 (0.685-7.067) | 0.820 (0.224-2.997) |
|  | < 5% | 2.268 (0.969-5.307) | 0.962 (0.364-2.544) |
|  | 5-9.9% | 1.383 (0.490-3.897) | 2.265 (0.572-8.975) |
| **Demographic** | |  |  |
|  | **Age** (years) (Ref = <45 y) |  |  |
|  | 65+ | **2.233 (1.018-4.898)** | **9.218 (2.559-33.19)** |
|  | 45-64 | 0.402 (0.139-1.161) | 1.865 (0.698-4.979) |
|  | **Gender** (Ref = Female) |  |  |
|  | Male | **2.632 (1.048-6.607)** | **4.075 (1.504-11.04)** |
|  | **Race** (Ref = Non-Hispanic White) |  |  |
|  | Others | **0.282 (0.127-0.623)** | 0.798 (0.271-2.350) |
|  | Non-Hispanic Black | 0.632 (0.237-1.687) | 0.848 (0.225-3.194) |
|  | **Education** (Ref = College or above) |  |  |
|  | Under 12th grade | 0.992 (0.421-2.335) |  |
|  | High school graduate | 0.826 (0.296-2.309) |  |
|  | **Marriage** (Ref = Married) |  |  |
|  | Never married | **2.695 (1.100-6.606)** | 1.344 (0.256-7.043) |
|  | Widowed/Divorced/Separated | 1.779 (0.726-4.356) | **2.740 (1.028-7.299)** |
|  | **Income ratio** (Ref =>3.50 (richest)) |  |  |
|  | 0.00-1.30 | 2.399 (0.763-7.537) |  |
|  | >1.30-3.50 | 2.531 (0.846-7.572) |  |
| **Lifestyle** | |  |  |
|  | **Smoking** (Ref = Non-smoker) |  |  |
|  | Smoker | **2.812 (1.233-6.414)** | 1.330 (0.468-3.779) |
|  | **Alcohol drinking** (Ref = Non-heavy drinker) |  |  |
|  | Heavy drinker | 1.897 (0.756-4.762) |  |
|  | **BMI** (Ref = Normal) |  |  |
|  | Underweight | 0.836 (0.181-3.864) |  |
|  | Overweight | 1.064 (0.394-2.868) |  |
|  | Obese | 0.895 (0.303-2.637) |  |
|  | **Physical activity** (Ref = Active) |  |  |
|  | Inactive | 1.439 (0.694-2.987) |  |
|  | **Sleep (hours)** (Ref =7-9) |  |  |
|  | < 7 | 1.542 (0.724-3.287) |  |
|  | > 9 | 1.926 (0.678-5.474) |  |
| **Comorbidities** (Ref =No) | |  |  |
|  | DM | 2.289 (0.877-5.973) |  |
|  | Hypertension | 1.996 (0.961-4.146) |  |
|  | CVD | 1.850 (0.804-4.254) |  |
|  | COPD | 0.589 (0.185-1.878) |  |
|  | Cancer | **2.666 (1.241-5.725)** | 2.774 (0.978-7.863) |
|  | Hearing impairment | 1.774 (0.372-8.464) |  |
|  | Depression | 1.778 (0.473-6.681) |  |
|  | CKD | **5.257 (2.316-11.93)** | **5.985 (2.528-14.16)** |
|  | Low social contact | 2.255 (0.728-6.983) |  |
|  | Anemia | 1.117 (0.494-2.526) |  |
| **Laboratory** | |  |  |
|  | NLR (Ref = < 3 (1000 cell/uL)) |  |  |
|  | NLR ≧ 3 | **3.901 (1.731-8.792)** | 1.535 (0.599-3.936) |
|  | HDL-C (Ref = ≧40 (mg/dL) |  |  |
|  | HDL-C < 40 | 0.688 (0.301-1.576) |  |
|  | Vitamin D in serum (Ref =75-125 (nmol/L)) |  |  |
|  | < 75 | 0.952 (0.411-2.202) |  |
|  | > 125 | 3.930 (0.625-24.71) |  |
| **Dietary/nutrition^+^** | |  |  |
|  | Protein (gm/day)^*^ | 1.017 (0.805-1.286) |  |
|  | Carbohydrate (gm/day)^*^ | **0.921 (0.874-0.971)** | **0.946 (0.901-0.993)** |
|  | Fiber (gm/day)^*^ | 0.578 (0.329-1.014) |  |
|  | Saturated fatty acid (gm/day)^*^ | 1.005 (0.487-2.071) |  |
|  | Magnesium (mg/day)^*^ | 0.985 (0.955-1.016) |  |
|  | Zinc (mg/day) | **1.018 (1.003-1.032)** | **1.024 (1.008-1.040)** |
|  | Vitamin B6 (mg/day) | 0.912 (0.692-1.203) |  |
|  | Vitamin B12(mcg/day) | 1.016 (0.994-1.038) |  |
|  | Vitamin C (mg/day)^*^ | 0.981 (0.930-1.034) |  |
|  | Vitamin D (mcg/day) | 0.985 (0.930-1.044) |  |
|  | Vitamin E (mg/day) | 0.971 (0.917-1.029) |  |
|  | Niacin (mg/day) | 0.989 (0.955-1.025) |  |
|  | Folate (mcg/day)^*^ | **0.979 (0.960-0.999)** | **0.961 (0.936-0.987)** |
|  | Selenium (mcg/day)^*^ | 0.945 (0.831-1.074) |  |
|  | Calcium (mg/day)^*^ | 0.995 (0.987-1.003) |  |

+ adjusted the energy intake of each nutrition to the benchmark of 2000 Kcal.

*Increased per 10 unit

BMI: Body mass index,

DM: Diabetes mellitus

CVD: Cardiovascular disease

COPD: Chronic obstructive pulmonary disease

CKD: Chronic kidney disease

NLR: Neutrophil to lymphocyte ratio

HDL-C: High-Density Lipoprotein Cholesterol

**Supplementary Figures**

**
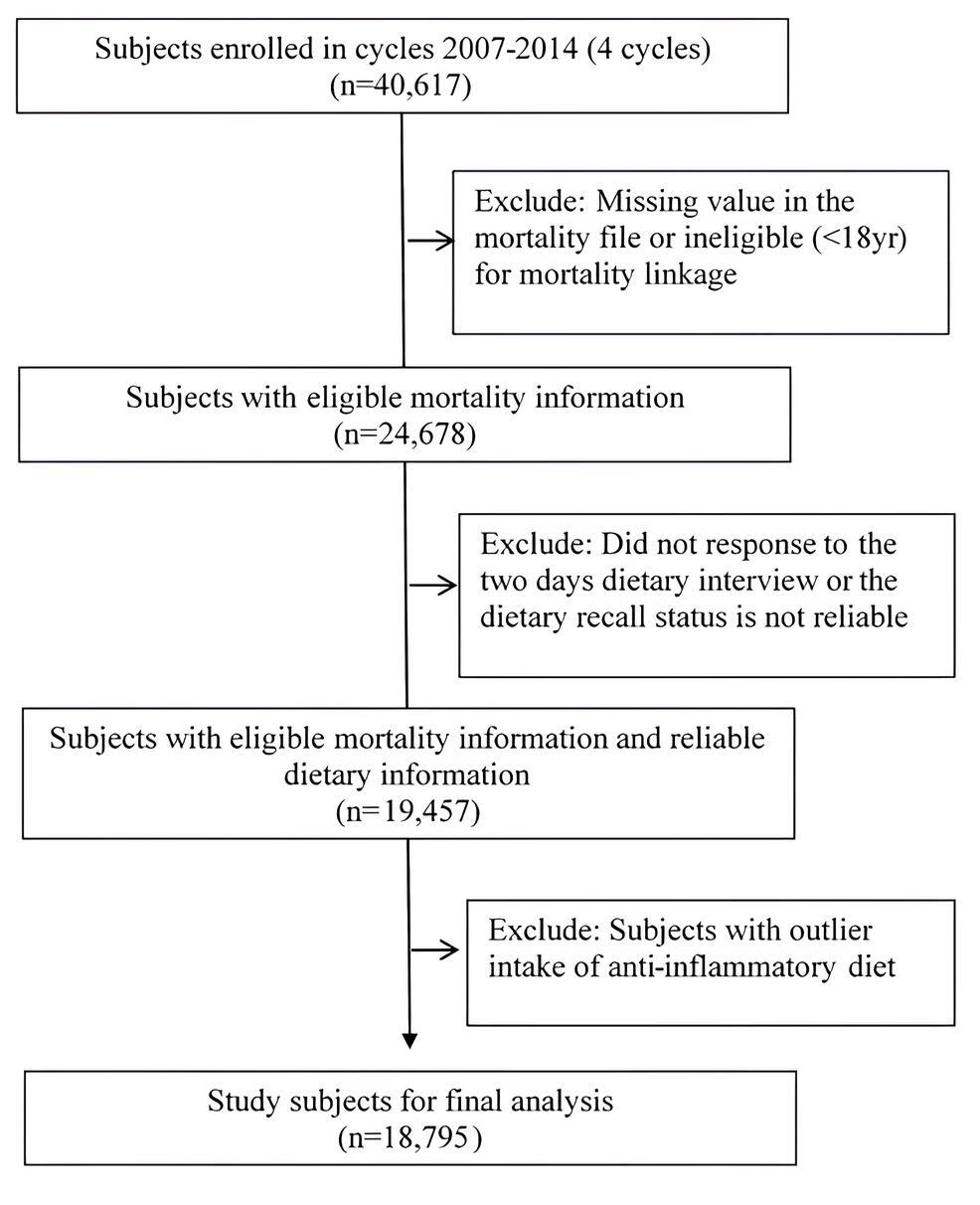
**

**Figure S1.** **Study cohort selection flowchart.** Flowchart illustrating the selection of 18,795 participants for the final analysis from the NHANES 2007–2014 cycles. Exclusions included incomplete dietary interviews (n = 5,221) and unreliable data (n = 6,661), ensuring a robust dataset for mortality risk assessment.

**
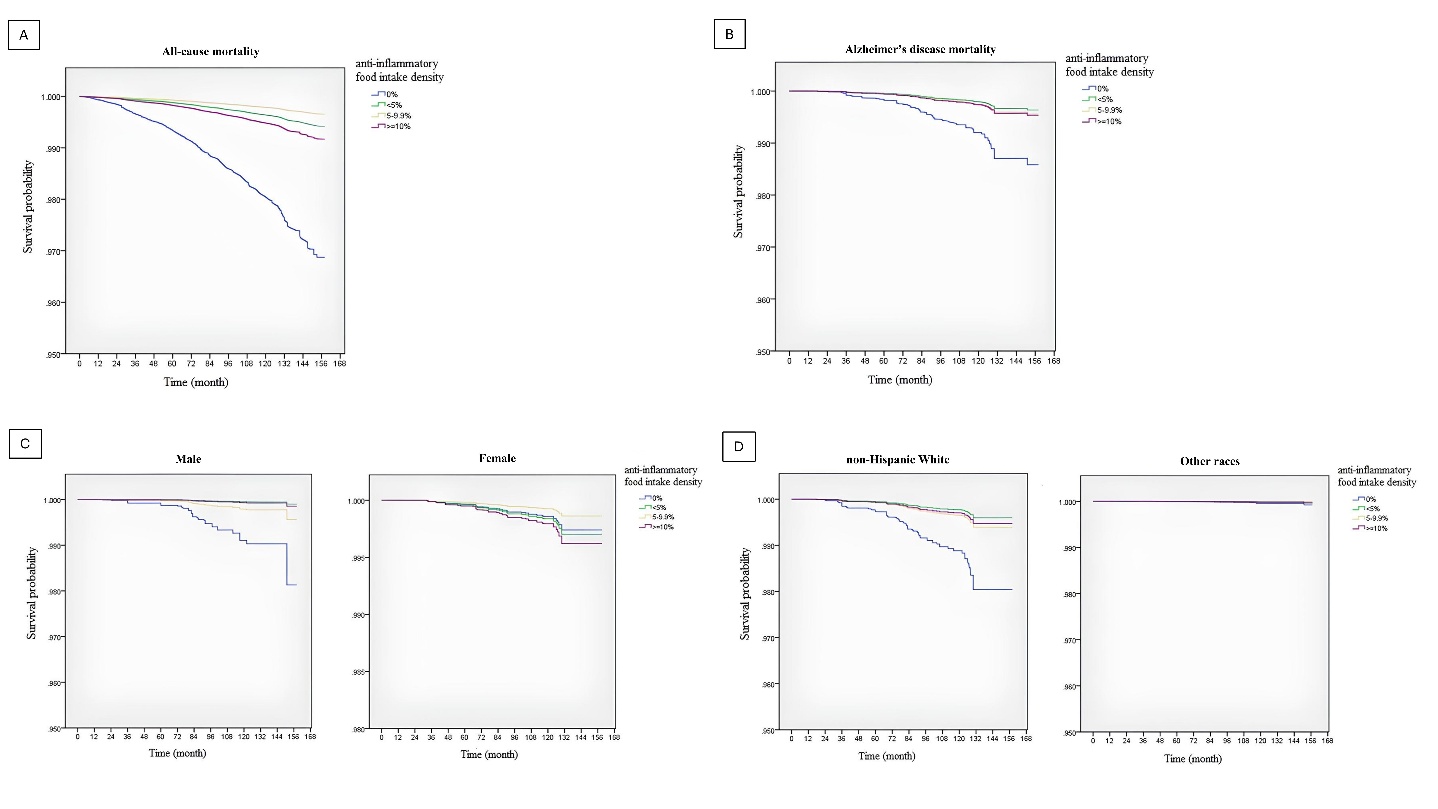
**

**Figure S2.** **Kaplan-Meier survival curves for mortality outcomes by anti-inflammatory food intake.** (A) Overall all-cause survival curves, demonstrating that participants with 0% anti-inflammatory intake exhibit the lowest survival probability, whereas those with ≥10% intake have the highest. (B) Overall Alzheimer’s disease (AD) survival curves among participants, showing a similar trend. (C) AD survival curves stratified by gender, with a pronounced survival disadvantage for males with 0% intake. (D) AD survival curves stratified by race/ethnicity, indicating that non-Hispanic White individuals with 0% intake had the lowest survival, while participants with moderate (5–9.99%) or higher intake fared better.
